# N-Terminal Acetylation Inhibits Protein Targeting to the Endoplasmic Reticulum

**DOI:** 10.1371/journal.pbio.1001073

**Published:** 2011-05-31

**Authors:** Gabriella M. A. Forte, Martin R. Pool, Colin J. Stirling

**Affiliations:** Faculty of Life Sciences, University of Manchester, Manchester, United Kingdom; University of California San Francisco and Howard Hughes Medical Institute, United States of America

## Abstract

Amino-terminal acetylation is probably the most common protein modification in eukaryotes with as many as 50%–80% of proteins reportedly altered in this way. Here we report a systematic analysis of the predicted N-terminal processing of cytosolic proteins versus those destined to be sorted to the secretory pathway. While cytosolic proteins were profoundly biased in favour of processing, we found an equal and opposite bias against such modification for secretory proteins. Mutations in secretory signal sequences that led to their acetylation resulted in mis-sorting to the cytosol in a manner that was dependent upon the N-terminal processing machinery. Hence N-terminal acetylation represents an early determining step in the cellular sorting of nascent polypeptides that appears to be conserved across a wide range of species.

## Author Summary

The eukaryotic cell comprises several distinct compartments, called organelles, required to perform specific functions. The proteins in these compartments are almost always synthesised in the cytoplasm and so require complex sorting mechanisms to ensure their delivery to the appropriate organelle. Of course, not all proteins need to leave the cytoplasm since many remain there to perform cytoplasmic functions. It is well known that many proteins are modified by acetylation of their amino-terminus at a very early stage in their synthesis. We have discovered a profound difference between the likelihood of such a modification on cytoplasmic proteins and on those destined for one of the major organelles, the endoplasmic reticulum (ER): whereas cytoplasmic proteins are typically acetylated, those bound for the ER are largely unmodified. Moreover, when specific ER proteins were engineered to induce their acetylation we found that their targeting to the ER was inhibited. Our data suggest that N-terminal acetylation is a major determinant in protein sorting in eukaryotes.

## Introduction

The mechanism of translational initiation dictates that eukaryotic proteins are synthesized with an amino-terminal methionine residue. In 80% of yeast proteins studied, the initiating methionine is removed to reveal a new amino-terminal residue [1], and some 50% of proteins have their amino-terminal residue acetylated [2],[3]. Hence rather few proteins possess an unmodified N-terminus. However, while N-terminal processing is widespread, its biological significance is not well understood. It has been suggested to contribute to differential protein stability and has recently been shown to function as a degron for certain cytosolic proteins [4],[5], while in a small number of cases the processed N-terminus is known to contribute directly to protein function [6]–[9].

Methionine cleavage is catalysed by methionine aminopeptidases (MetAPs) that act co-translationally as the N-terminus emerges from the ribosome [1],[10]. MetAPs exhibit substrate specificity and are strongly influenced by the residue at position 2 (P2), with cleavage favoured by P2 residues with small side chains such as glycine, alanine, or serine [11],[12]. Yeast and humans each possess two MetAPs (MetAP1 & 2), and while yeast can tolerate the loss of either enzyme, the double mutant is lethal demonstrating that methionine processing is a vital function [13]. Interestingly, MetAP2 is the target for the potent anti-angiogenic compound fumagillin that exhibits anti-tumourigenic properties [14],[15].

Protein N-termini can also be modified by acetylation of the free α-amino group by N-α-acetyl transferases (NATs). Five distinct NATs have been identified with different substrate specificities. NatA normally acetylates N-terminal G, S, A, and T residues exposed by MetAP cleavage, whereas NatB acetylates methionine residues that are followed by either D, E, or N at P2 [3],[16],[17]. NatC acetylates certain methionines with either L, I, W, or F at P2, but other sequence elements influence processing in this case [18]. NatD appears to be specialised for histone N-acetylation [19] and finally NatE acetylates substrates with Leucine at P2 and Proline at P4 [20].

While most proteins remain in the cytoplasm after synthesis, others are targeted to different compartments. Those destined for the secretory pathway typically possess an N-terminal signal-sequence which directs them to the endoplasmic reticulum (ER) [21]. These proteins are translocated into the lumen of the ER, via the Sec61 translocon, whereupon their signal-sequence is removed by signal peptidase [22]. A subset of membrane proteins can be targeted to the ER via non-cleaved internal signal anchor or C-terminal trans-membrane segments, which act as both targeting and membrane-integration signals.

N-terminal signal sequences are degenerate in primary structure but are typically 15–30 residues long, and usually comprise charged/polar residues, followed by 6–15 hydrophobic residues and a polar C-terminal region containing the cleavage site for signal peptidase [23],[24].

In yeast, there are two pathways by which secretory proteins are targeted to the ER. The co-translational pathway is mediated by Signal Recognition Particle (SRP), which recognises a signal sequence emerging from the ribosome and targets the ribosome-nascent chain (RNC) complex to the translocon via SRP-receptor (SR) [25],[26]. The targeted ribosome then binds tightly to the cytosolic surface of Sec61p allowing the elongating polypeptide chain to be delivered directly into the translocation channel [27]–[29]. The alternative “post-translational” pathway is independent of SRP/SR [30] and targets full-length polypeptides in a reaction that requires cytosolic chaperones that maintain precursors in a translocation-competent conformation [31]–[33]. Translocation occurs via the same Sec61-channel, but in this case, targeting requires the essential integral membrane protein Sec62p that interacts with precursor and may constitute a specific receptor [34]. Mammalian cells possess a homologue of SEC62, but this mode of translocation remains poorly characterized in metazoans [35],[36].

Properties of the signal sequence, and in particular the hydrophobicity of the central core, determine which pathway a substrate will access, with more hydrophobic signal sequences utilizing the SRP pathway [30].

Cleavage of the signal sequence reveals a novel N-terminus for the mature translocated protein, which is located in the ER lumen and so inaccessible to the N-terminal processing enzymes. The processing status of the initiating methionine of signal sequences has largely been ignored, particularly as such N-termini are not detected in proteomic analyses. We therefore decided to investigate the N-terminal processing of signal sequences using a combination of bioinformatic and experimental approaches and find that N-terminal modification is incompatible with targeting to the ER.

## Results

**Figure 1:**
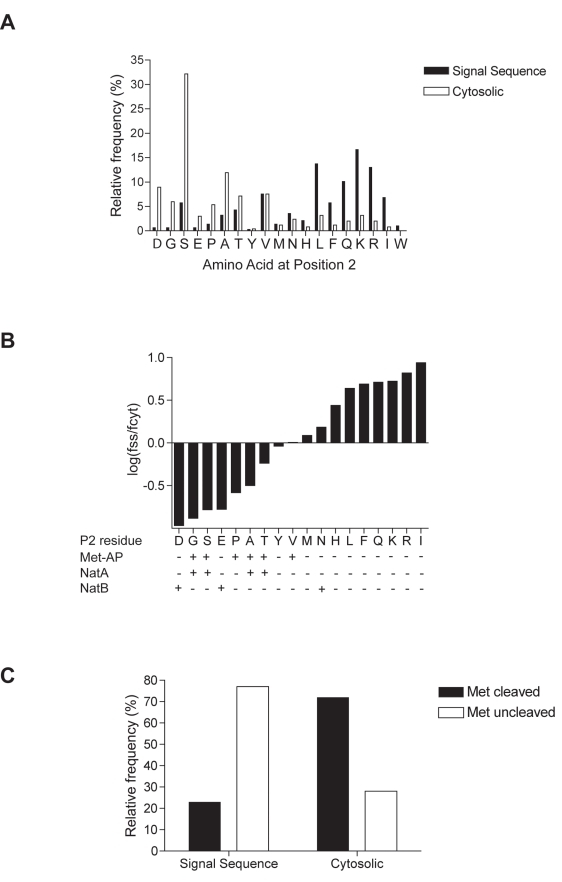
Amino acid frequency at P2 of signal sequences versus cytosolic proteins. (A) Relative frequency of amino acids at P2 of a filtered set of 277 signal sequence-containing proteins from *S. cerevisiae* was compared to a similar size group (*n* = 252) of randomly selected cytosolic proteins. Frequency distribution between the groups differed significantly (*p*<0.0001, χ^2^ = 207.3 18 *df*). (B) Ratio of relative frequency of P2 residues between signal sequence (*f_ss_*) and cytosolic (*f_cyt_*) proteins. Tryptophan was absent from the cytosolic group; therefore, no log(*f*_ss_/*f*_cyt_) value is plotted. P2 specificities of MetAP, NatA, and NatB are indicated. (C) Predicted methionine cleavage of signal sequence and cytosolic N-termini based on relative P2 frequency. For complete datasets, see Tables S1–S4.

Signal sequence recognition and N-terminal processing both occur co-translationally as the nascent chain emerges from the ribosome [10],[37]. We therefore decided to investigate whether secretory proteins might be subject to N-terminal processing in a similar manner to their cytosolic counterparts. As the P2 residue is the major determinant of N-terminal processing, we first surveyed the amino acid frequency at this position for signal sequence-containing proteins versus cytosolic proteins (Figure 1A). Surprisingly, we found a significantly different frequency distribution between the two sets (*p*<0.0001, according to the χ^2^ test with 18 degrees of freedom). Lysine, leucine, and arginine were most frequent at P2 in signal sequences but were rarely found at this position in the cytosolic set. Conversely, while serine and alanine were most frequent at P2 in cytosolic proteins, these were less evident in signal sequences. A clear pattern emerged when the ratio of frequencies were compared between the two classes of proteins (Figure 1B); small and acidic residues were strongly biased towards cytosolic proteins, whereas large and basic ones were favoured in signal sequences. The frequency of small residues at P2 in cytosolic proteins predicts that ∼72% of these proteins would be substrates for MetAP cleavage (Figure 1C), in good agreement with empirical data from proteomic studies [2]. In contrast only 23% of signal sequences would be predicted to be MetAP substrates (Figure 1C). Hence our data reveal that for signal sequences there appears to be a strong selection for P2 residues that would maintain the original N-terminal methionine.

**Figure 2:**
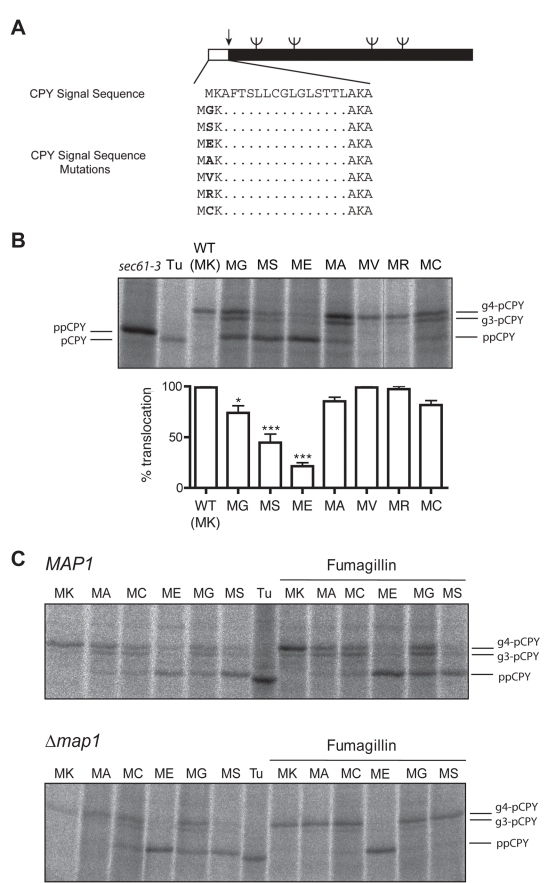
Removal of the N-terminal methionine inhibits ER translocation of CPY. (A) Schematic of wild-type CPY and P2 mutants. Signal peptide sequence, position of N-glycosylation (ψ), and signal peptidase cleavage (↓) sites are indicated. (B) Yeast cells (*Δpep4,Δprc1*) expressing either wild-type or mutant CPY were pulse-labelled with [^35^S]methionine/cysteine, then CPY immunoprecipitated, and analysed by SDS-PAGE and phosphorimaging. Positions of glycosylated CPY (g4-pCPY and g3-pCPY) are indicated as are the untranslocated ppCPY and signal-sequence cleaved, non-glycosylated CPY (pCPY) observed in *sec61-3* cells and in tunicamycin-treated wild-type cells (Tu), respectively. Translocation efficiency was determined by quantification of ppCPY and g3- and g4-pCPY from three independent experiments. Error bars represent standard error of the mean. Asterisks represent *p*<0.05 (*) and *p*<0.001 (***) according to the one-way analysis of variance with Tukey's multiple comparison test. (C) CPY translocation was analysed as in (B), in a wild-type (*Δpep4,Δprc1*) and isogenic *Δmap1* strain in the presence and absence of the Map2 inhibitor fumagillin (for quantification, see Figure S1).

We next addressed whether this bias was of functional significance for ER translocation. The signal sequence of Carboxypeptidase Y (CPY) [38] begins with “MK” and so, like most secretory proteins in our analysis, is predicted to remain unprocessed. Rather than mutating the native P2 residue we chose to insert one of seven different amino acids between the initiator methionine and the following lysine residue (Figure 2A). We then assessed the translocation efficiency of these mutants in vivo by monitoring their ER-dependent glycosylation (Figure 2B). Insertion of arginine or valine had no effect on the efficiency of translocation, demonstrating that an insertion at this position does not inherently perturb signal sequence function. However, the other five insertions tested all resulted in translocation defects indicated by the accumulation of the cytosolic precursor form of preproCPY (ppCPY). The most significant defects were observed for glycine, serine, and glutamate, which are three of the four residues most biased in their frequency distribution towards cytosolic proteins (Figure 1B). Thus the bias observed in our bioinformatic analysis correlates with defects in translocation, thereby implying an important role for P2 in a functional signal sequence.

The inhibitory effects of these various P2 residues might reflect either some simple perturbation of the signal sequence or their predicted impact on N-terminal processing. We reasoned that if processing alone were responsible for the effects, then inhibiting MetAP activity might restore translocation of the mutant proteins. We therefore analysed translocation in wild-type and Δ*map1* cells in the presence of the Map2p inhibitor fumagillin (Figures 2C and S1). In wild-type (*MAP1*) cells, fumagillin had little or no effect on the translocation of native (MK) CPY nor the translocation defects observed for the various insertion mutants. Similarly, the absence of Map1 alone (*Δmap1*) had no discernible effect on any of the translocation substrates. In contrast, when *Δmap1* cells were treated with fumagillin we found almost complete restoration of translocation for the MA, MC, MG, and MS mutants. All four are predicted substrates for Met-cleavage, and our data demonstrate that their inhibitory effects are entirely dependent upon MetAP activity. In contrast, ME is not a substrate for MetAP and we found that the translocation defect for this mutant persisted under these conditions. The effect of fumagillin was therefore substrate-specific, correlating precisely with the known specificity of MetAPs [11]. We therefore conclude that MetAP-dependent cleavage of a signal peptide's initiating methionine has a strong inhibitory effect on the translocation of CPY.

**Figure 3:**
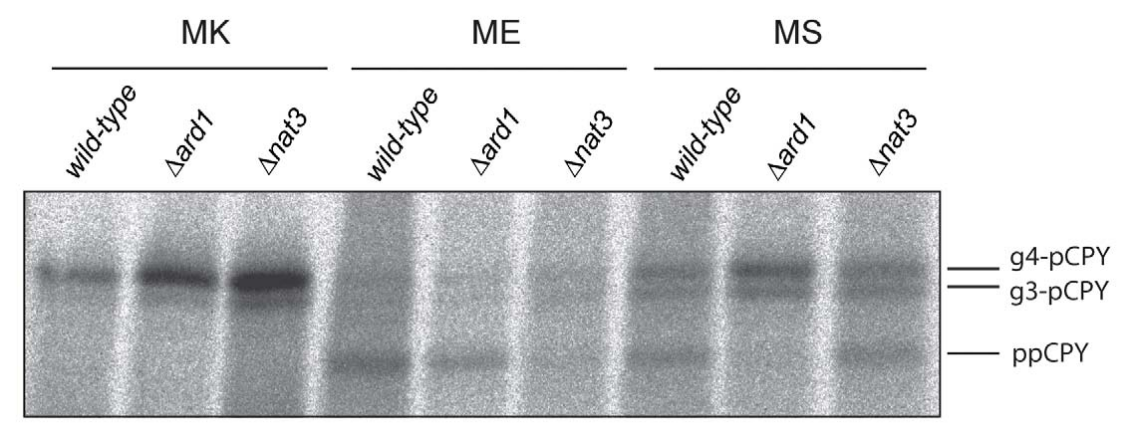
N-terminal acetylation blocks protein translocation. Translocation of wild-type, MS, and ME mutants of CPY was examined z(as in Figure 2B) in wild-type and *Δard1* and *Δnat3* strains, which lack NatA and NatB activity, respectively. Data are representative of three independent experiments.

In our analysis, the ME and MS mutations had the strongest effects on translocation (Figure 2B) and these P2 residues displayed extreme bias against their occurrence in natural signal sequences (Figure 1B). While “ME” is not a substrate for MetAP, it is known to promote N-α-acetylation of the N-terminal methionine by NatB [6]. Likewise, the P2 serine, once revealed by MetAP, is predicted to be N-α-acetylated by NatA. We therefore tested whether acetylation might be the key determinant affecting translocation by analysing translocation efficiencies in either NatA(Δ*ard1*) or NatB(Δ*nat3*)-deficient strains (Figure 3). In Δ*ard1* cells, translocation of MS-CPY appeared largely restored while the ME mutant remained unaffected. The converse was observed in the Δ*nat3* strain. Importantly, the ability of the different *Nat* mutants to rescue precursor translocation matched precisely the substrate specificities of NatA and NatB for MS and ME, respectively. Moreover, the observation that inhibition of MAP activity specifically rescues the translocation of NatA substrates is entirely consistent with methionine cleavage being a prerequisite for NatA-dependent acetylation. Thus, it is the N-α-acetylation of these substrates that is the major determinant in the inhibition of translocation in vivo.

**Figure 4:**
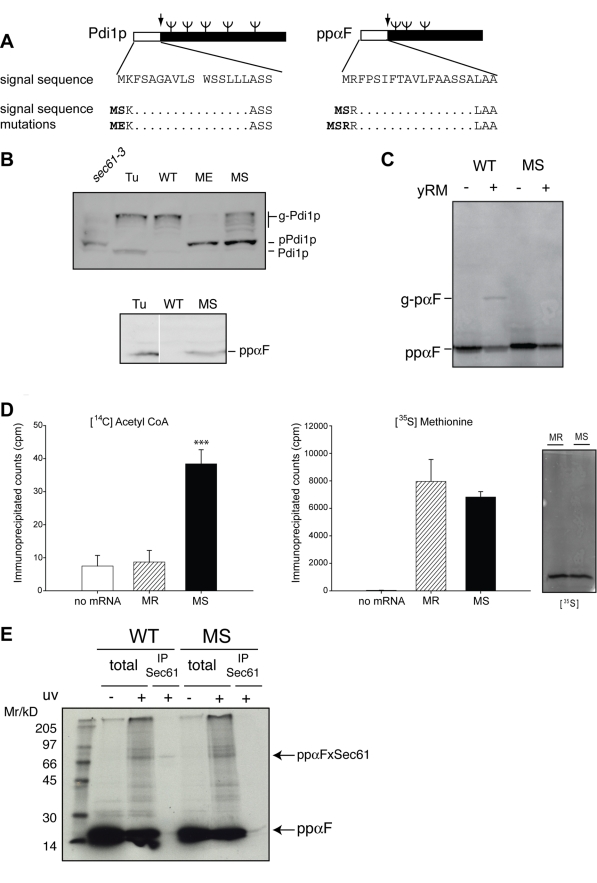
Protein N-acetylation inhibits ER translocation both in vivo and in vitro. (A) Schematic of wild-type and P2 signal sequence mutants of Pdi1p and preproα-factor. Position of N-glycosylation (ψ) and signal peptidase cleavage (↓) sites are indicated. (B) Wild-type and indicated mutants of myc-tagged Pdi1p and ppαF were expressed in wild-type (*Δpep4*) or *sec61-3* strains, and treated, where indicated, with Tunicamycin (Tu). Steady-state levels of protein were determined by preparation of cell extracts from these strains and analysis by Western blot with anti-myc antibodies. (C) Wild-type (MR) and MS forms of lysine-less ppαF (where all lysines had been mutated to arginine) were translated in vitro, then incubated with yeast microsomes (yRM). Position of non-translocated (ppαF) and signal-sequence cleaved, glycosylated (g-pαF) are indicated. (D) Lysine-less forms of both wild-type (MR) and MS ppαF were translated in vitro in the presence of either [^35^S] methionine or [^14^C] acetyl-CoA and immuno-precipitated with anti-ppαF antibodies before analysis by either scintillation counting or SDS-PAGE. Error bars represent standard deviation; three asterisks indicate *p*<0.001 according to the two-tailed student's *t* test. (E) Wild-type (MR) and MS ppαF with lysine residues at positions 5 and 12 were translated in vitro in the presence of [^35^S] methionine and TDBA-lysyl-tRNA. Targeting to microsomes was performed in the absence of ATP and then cross-linking induced by uv-irradiation. Where indicated, samples were denatured and immuno-precipitated with Sec61 antisera.

We next examined the effect of mutants predicted to induce acetylation of two independent ER translocation substrates, namely Pdi1p and prepro-alpha factor (ppαF) (Figure 4A and 4B). The signal sequence of Pdi1p begins MK and hence is not predicted to be a substrate for MetAP or N-acetylation [2]. MSK and MEK mutations both led to accumulation of non-translocated precursor and a reduction of fully translocated glycosylated Pdi1p at steady state. Furthermore, analysis by mass-spectrometry confirmed that the MSK mutant of pPdi1 was methionine-processed and N-acetylated in vivo, as predicted (Figure S2). No peptides corresponding to an unmodified N-terminus were detected.

Wild-type ppαF, which begins MR, is efficiently translocated and secreted. In contrast an MS mutant, which is a predicted substrate for NatA, accumulated in cells, as the non-translocated precursor. Hence, the inhibitory effect of acetylation appears widespread and not restricted to CPY.

Next we sought to reconstitute this phenomenon in vitro using ppαF. We translated both wild-type (MR) and MS mutant forms of ppαF in reticulocyte lysate and then incubated these precursors with yeast microsomes (Figures 4C and S3). We observed microsome-dependent translocation and glycosylation of wild-type ppαF but found no evidence of translocation of the MS mutant. Thus the inhibitory effect of the P2 Serine can also be reconstituted in vitro.

Our data thus far indicate that MS-ppαF would be acetylated following processing by MetAP. To verify this directly we performed in vitro translations in the presence of 1-[^14^C]-acetyl-CoA and detected incorporation of radiolabel into MS-ppαF but not wild-type (Figure 4D). For this experiment, we utilised a ppαF variant where all lysines have been mutated to arginine; hence, the only primary amine potentially available for acetylation is the N-terminal αNH_2_ group. These in vitro data demonstrate directly that the MS mutant form of ppαF is indeed acetylated as predicted and support our hypothesis that N-terminal acetylation inhibits ER translocation.

Charge distribution across the signal sequence has been shown to affect translocation efficiency [39]. N-α-acetylation of the signal peptide would reduce the overall positive charge of the N-terminus by +1, and therefore one potentially trivial explanation might be that it is the loss of positive charge, rather than acetylation per se, that inhibited translocation. However, we can exclude this possibility given that the insertion of an additional arginine residue at position 3 (MSRR), which restores the overall charge of the N-region following N-α-acetylation, also failed to translocate (Figure S3).

We next wished to assess the stage at which the translocation of an acetylated MS substrate is blocked. We incubated in vitro translated wild-type (MR) ppαF with yeast microsomes in the absence of ATP, which permits targeting to Sec61, but not subsequent translocation. Using site-specific photocross-linking probes incorporated into the signal sequence, we could detect a complex spectrum of uv-induced adducts as has been reported previously (Figure 4E; [40]). An adduct of ∼50 kD could be readily immunoprecipitated with Sec61p antisera, indicating the engagement of precursor with the translocon. In striking contrast, the MS mutant completely failed to crosslink with Sec61p. Hence we conclude that targeting arrests at a step prior to the interaction of the precursor with the translocon.

**Figure 5:**
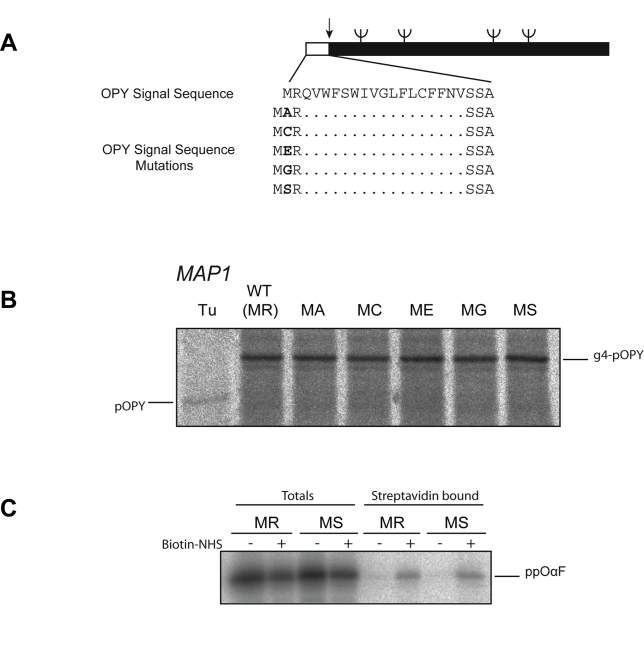
An SRP-dependent precursor is refractory to N-acetylation. (A) Schematic of wild-type OPY (CPY with the endogenous signal sequence replaced by that of Ost1) and corresponding P2 signal sequence mutants. (B) Wild-type and mutant OPY translocation in vivo was monitored by pulse-labelling and immunoprecipitation as in Figure 2B. (C) Lysine-less wild-type (MR) and MS opαF (ppαF with the signal sequence replaced with that of Ost1p and all lysines mutated to arginine) were translated in vitro in the presence of [^35^S] methionine, denatured, and modified with amine-reactive sulfo-NHS-SS-biotin. Biotinylated proteins were re-isolated on immobilized-streptavidin and analysed by SDS-PAGE and phosphorimaging.

There are two pathways by which secretory precursors can be targeted to the ER; some precursors follow a post-translational Sec62p-dependent pathway, while substrates with more hydrophobic signal sequences utilise a co-translational SRP-dependent mechanism [25],[30]. As CPY, Pdi1p, and ppαF are all translocated post-translationally, we therefore sought to compare the behaviour of an SRP-dependent substrate. We chose the well-characterised SRP-dependent substrate OPY, a variant of CPY in which the endogenous signal sequence is replaced with that of Ost1p [41]. The OPY signal sequence begins MR, and so should remain unprocessed, enabling us to perform a precisely parallel mutational analysis to that for CPY (see Figure 2C). In striking contrast to CPY, we found that the introduction of various processable residues at P2 had no effect on the translocation of OPY (Figure 5A and 5B). Thus the observed inhibitory effect of an MS mutation on translocation can be suppressed in the context of an SRP-dependent signal sequence. This property was not limited to the Ost1p signal sequence; co-translational translocation of the SRP-dependent substrate D_HC_-αF [30],[42] into yeast microsomes using a yeast translation extract was also unaffected by the incorporation of a potentially acetylatable serine residue at P2 (Figure S4). Moreover, the well-characterized SRP-dependent substrates Sec71 and Dap2 (DPAP B) [30],[43] have P2 residues of S and E, respectively, entirely consistent with our finding that NAT substrates can be tolerated by the SRP pathway.

These data suggest either SRP can successfully target an acetylated substrate or alternatively such substrates might not be processed as expected. Therefore, to address this point we assessed whether or not the Ost1p signal sequence was N-terminally processed. We tested the MS mutant for the presence of any unmodified N-termini using a biotinylation assay to detect free α-NH_2_ groups in a protein completely lacking lysine residues. We observed no difference in the efficiency of biotinylation between wild-type (MR) and mutant (MS) suggesting that in the context of an SRP-dependent signal sequence, and contrary to expectation, the MS amino-terminal was not acetylated (Figure 5C).

This effect of SRP might go some way to explain the small, but not insubstantial, minority of secretory proteins predicted to be processed in our bioinformatic analysis. Consistent with this idea, we found that average peak hydrophobicity of signal sequences among this minority was significantly greater than for the majority subset of sequences (Figure S5). Overall, more than 99% of signal sequences were either not predicted to be acetylated or were sufficiently hydrophobic to interact with SRP.

**Figure 6:**
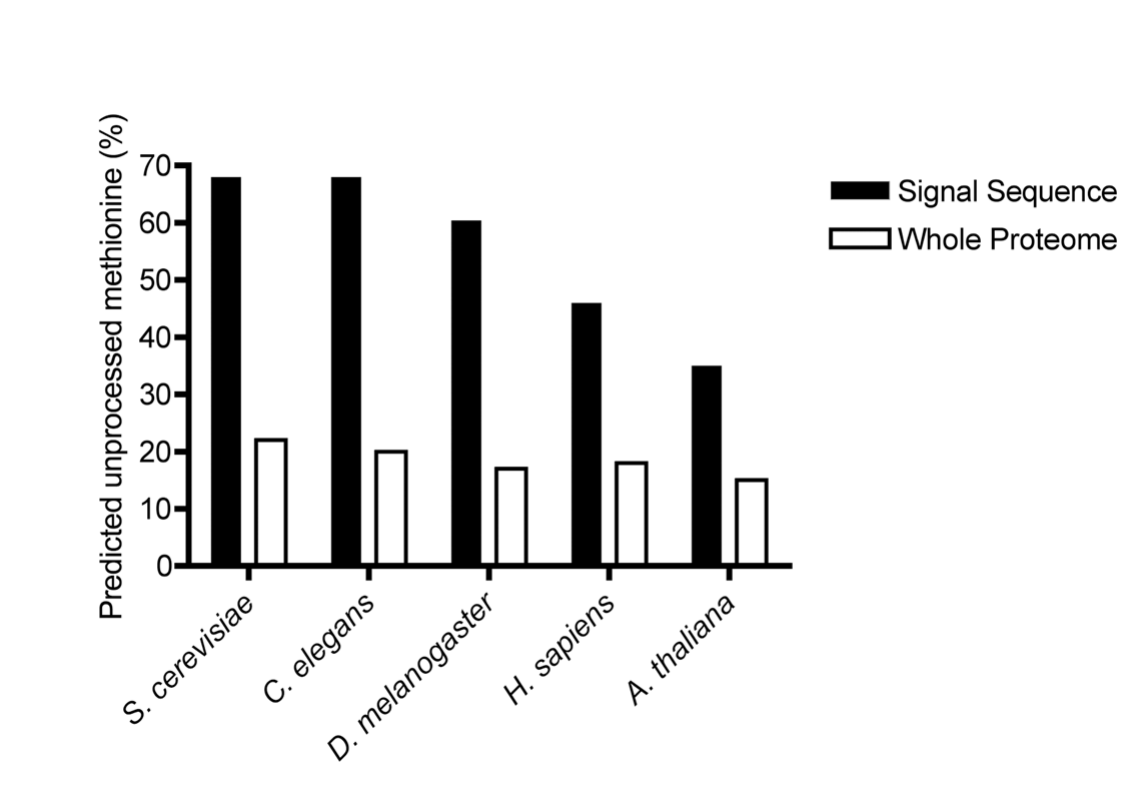
A bias against N-terminal processing of signal sequences is conserved across eukaroytes. Predicted frequency of an unprocessed initiating methionine in signal sequences from *S. cerevisaie* (*n* = 277), *C. elegans* (*n* = 378), *Drosophila* (*n* = 448), human (*n* = 595), and *Arabidopsis* (*n* = 500) compared to the respective proteomes as a whole [2]. For complete datasets, see Tables S5 and S6.

Having validated the biological significance of the bias observed in our bioinformatic study, we extended our analysis from yeast to higher eukaryotes (Figure 6). The pattern observed in nematodes and insects was remarkably similar to that seen in yeast, with ∼70% of signal peptides predicted to retain an unprocessed methionine compared to only 20% for the proteome as a whole [2]. The trend was similar in humans and plants, albeit less pronounced, with ∼50% of secretory N-termini predicted to remain unprocessed compared to 15% for the proteome as a whole [2]. Thus this phenomenon appears not to be restricted to fungi but is very widely conserved.

## Discussion

Here we describe the striking observation that yeast signal sequences display a profound bias against N-terminal processing. The bias is precisely converse to that observed in cytosolic proteins where N-terminal processing is highly favoured. Moreover, we show that this bias is of functional significance as introduction of residues at position 2 which promote N-terminal processing inhibits translocation in to the ER. Importantly this inhibition can be reversed by blocking N-terminal processing, confirming that it is the processing itself that leads to the block in translocation. The bias against N-terminal processing is not restricted to yeast but is also observed across eukaryotes, suggesting this is a widely conserved phenomenon.

It is possible that other factors distinct from N-terminal processing might affect the observed bias in amino acid frequency at position 2. We considered the potential effect of the Kozak consensus sequence that favours a G at the +4 position (corresponding to the first base of codon 2) in genes optimised for translation efficiency [44]. However, while this might contribute to the bias observed among cytosolic proteins, it is unlikely to be the dominant feature since it does not explain the predominance of Serine at position 2. Furthermore, the Kozak consensus does not have such a strong effect in yeast and it has recently been reported that the effect of the +4 position may be more important in promoting N-terminal modification than in influencing initiation efficiency [45].

A second possible factor influencing the P2 frequency distribution could be the previously reported bias for an adenine-free stretch within the signal-sequence coding region of a secretory mRNA, which is important for its nuclear export [46]. However, this also seems an unlikely explanation as lysine, with its A-rich codon (AAA/AAG), is actually more frequent at position 2 of signal sequences as compared to cytosolic proteins. Critically, however, both translation initiation and nuclear mRNA export operate independently of N-terminal processing and so would not lead to translocation defects that could be reversed by N-terminal processing mutants, as we observe. Furthermore the restoration of translocation in such processing mutants shows a precise substrate dependency, ruling out rescue of translocation by some indirect effect. Hence, while we would not completely exclude a minor role for the Kozak consensus or mRNA elements in influencing the P2 residue of signal sequences, the strong correlation and clear functional effects make a bias against N-terminal processing the simplest and most likely explanation of the relative P2 residue frequency.

A trivial explanation for the inhibitory effect of acetylation could be the change in charge distribution across the signal sequence, which is known to be important for targeting [39]. However, this appears unlikely, firstly as insertion of an additional positively charged residue to counteract the loss of the +1 charge following acetylation of the free amino terminus did not restore translocation (Figure S3). Secondly, translocation of the ME CPY mutant can be restored in a strain lacking NatB activity (*Δnat3*), which results in the same net N-terminal charge as is present in the acetylated MS CPY mutant, which fails to translocate (Figure 3). Hence simple charge distribution alone cannot explain the inhibitory effects of N-acetylation.

Overall our data indicate that N-acetylation inhibits ER translocation and that most secretory proteins avoid this by virtue of a P2 residue that prevents processing. Interestingly, SRP-dependent substrates appear to evade this effect as SRP blocks N-terminal N-acetylation even in the presence of a P2 residue predicted to be a NatA substrate. SRP and NatA are both thought to contact the ribosome via the same site (ribosomal protein Rpl25/L23a) [47]–[49]. Hence competition for this site would provide a potential mechanistic explanation for this phenomenon. This finding also predicts that while the P2 residue is the major determinant of N-acetylation by NatA, there are scenarios where N-acetylation does not occur, despite the presence of an appropriate P2 residue. Empirical evidence for this prediction was recently provided by the global analysis of N-acetylation of the drosophila proteome [50].

Comparison of predicted N-terminal processing of signal sequences across other species indicates an almost identical bias for nematodes and drosophila as seen in yeast. In plants and humans, the bias is still present but is less marked. Interestingly, a bias against predicted N-terminal processing (73%) has also been noted for prokaryotic signal sequences [51]. Hence the bias against processing of signal sequences appears widespread and not restricted to yeast.

Current dogma suggests that the SRP-dependent targeting pathway is more pervasive in mammals. As SRP appears to allow substrates to evade the effects of acetylation, this may well explain why the bias against N-terminal processing is less pronounced in humans. Nevertheless, homologues of the SRP-independent pathway components Sec62 and Sec63 are present in mammals and form complexes with the Sec61 translocon [35],[36]. Furthermore, both mammalian and drosophila Sec62 can functionally replace their yeast counterpart [52],[53]. These observations, combined with our observed bias against N-terminal processing in these organisms, suggest that although SRP-dependent targeting is perhaps more dominant, Sec62-dependent translocation still likely occurs. Identification of substrates for this pathway remains an important question to be addressed in the future.

What might be the reason as to why secretory and cytosolic proteins have a precisely converse bias for N-acetylation? Cytosolic proteins, once synthesized, typically fold rapidly to their final tertiary structure in the cytoplasm. In contrast, secretory precursors must reach the translocon in an unfolded state in order to be competent for translocation. Post-translationally translocated substrates achieve this by their interactions with cytosolic chaperones that prevent their folding within the cytoplasm [32]. SRP-dependent substrates are targeted co-translationally and so reach the translocon as short nascent chains, thus eliminating the possibility of folding in the cytoplasm. It is not known what causes translocation substrates to recruit these chaperones, but our data allow us to propose a model in which acetylation determines the fate of nascent polypeptides. We speculate that acetylation identifies nascent polypeptides, very early in their synthesis, as being destined to fold in the cytoplasmic compartment. Most secretory proteins are unmodified and so would be delayed in their folding sufficiently to facilitate their functional interaction with the translocon. This would be entirely consistent with our finding that acetylation blocks secretory substrate interaction with Sec61, arresting the protein in the cytosol.

Not all proteins that fold and remain in the cytosol are acetylated. It may be that such modification would be incompatible with function, but it might also be that such proteins have more complex folding requirements; for example, they might be required to fold more slowly, perhaps relying on the recruitment of specific cytosolic chaperones.

An alternative biological explanation for this phenomenon could relate to a proofreading step for Sec62-dependent substrates. Unlike their SRP-dependent counterparts, Sec62-dependent signal sequences are only modestly hydrophobic [30]. It is quite likely, therefore, that globular cytosolic proteins may contain internal regions of similar hydrophobicity, which upon folding form the hydrophobic core of such proteins. Clearly, it is critical that these proteins do not translocate into the ER and become mis-sorted. Entirely consistent with this idea, it has been shown that randomly selected regions of the mature domains of both CPY and invertase (Suc2) can promote translocation, albeit inefficiently, when positioned at the N-terminus [54],[55].

A requirement for a free N-terminus proximal to the hydrophobic region could provide a mechanism to prevent internal regions of non-secretory proteins engaging the translocation machinery. Modification of the N-termini of cytosolic proteins would also help prevent mis-sorting.

Internal ER targeting sequences of course exist, but they tend to be trans-membrane domains which act as signal anchor sequences; hence they are much more hydrophobic and thus promote targeting via the SRP pathway [30].

In summary, our finding that N-terminal processing inhibits ER translocation of secretory proteins identifies a non-acetylated N-terminus as a hitherto unappreciated yet general feature of signal sequences, which is necessary to promote efficient targeting of substrates to the ER translocon.

## Materials and Methods

### Bioinformatics

The set of *S. cerevisiae* signal sequence-containing proteins was obtained from the signal peptide database (SPdb) v 5.1 [56]. This set of 291 sequences was manually filtered for duplicates, dubious ORFs (as defined by SGD), and proteins known to be localized to mitochondria, to yield a final filtered set of 277 ORFs. For a complete list of ORFs, see Table S1. The P2 amino acid frequency distribution did not differ significantly between the filtered and unfiltered sets (χ^2^ = 5.17, 19 *df*). Graphical and statistical analysis was performed using Prism 4.0 (GraphPad). MetAP cleavage was assumed for P2 residues A, C, G, P, S, V, and T [11],[12]. The yeast cytosolic dataset (Table S2) was generated by random selection from SGD of proteins with known cytosolic localization. Prediction of N-acetylation was performed as described previously [2]; where appropriate, the P3 residue was also taken into consideration. MN, which is only predicted to lead to N-acetylation in 55% of cases [2], was scored as acetylated. Human and *Caenorhabditis elegans* signal sequence datasets were also obtained from the signal peptide database (SPdb) v5.1 [56]. *Drosophila melanogaster* and *Arabidopsis thaliana* datasets were obtained from the signal peptide website (www.signalpeptide.de, accessed March 2010). Peak hydrophobicity was determined by Kyte-Doolittle using a window size of 11 [30],[57].

### Yeast Strains

Yeast strains in this study are listed in Table S7. GFY3 was constructed by mating *Δpep4* and *Δprc1* strains, sporulation of the diploid, and selection of tetrads, which had three G418-resistant spores; spores were scored for null mutations by PCR and western blotting. GFY7 was made by PCR amplification of the pFA6a-His3MX6 module [58] with appropriate primers (Table S8); the PCR product was used to transform GFY3 and His^+^ colonies selected. GFY11 and GFY12 were made by PCR amplification of pAG26 [59] with appropriate primers (Table S8); the PCR products were used to transform Δ*prc1* followed by selection on Hygromycin B. All deletions were confirmed by PCR. Yeast strains were grown in either YPD (1% yeast extract, 2% peptone, and 2% glucose) or YNB (0.67% yeast nitrogen base, 2% glucose, and appropriate supplements) at 30°C, with the exception of pulse-labelling of MWY63 (*sec61-3*), which was grown at 30°C, then shifted to 17°C for 2 h.

### Plasmid Construction

The constructs which express ppCPY and ppOPY with position 2 insertion mutations of the signal sequence listed in Table S9 were made using the respective pairs of primers (Table S8) to perform site-directed mutagenesis of pMW346 or pOPY, respectively. pGF22, the *Psi*I/*Sph*I fragment of pA11-k5, was cloned into pEH3 to replace this portion of wild-type ppαF and thus making a lysine-free ppαF. pGF24 and pGF25 were constructed by PCR (Table S9) of the Ost1 signal sequence from pOPY and pOPY-S, respectively. The PCR products were digested with *Eco*RI/*Hinc*II and cloned into pGF23 (Table S9) to replace the ppαF signal sequence with that of Ost1 or the serine mutant version, respectively. *PDI1* was amplified from genomic DNA with appropriate primers (Table S8) that introduce a single C-terminal c-myc-tag. The PCR products were digested with *Psi*I/*Bam*HI and were then ligated into *Bst*Z171/*Bam*HI sites of pMW346, placing the *PDI1-myc* ORF under the control of the *PRC1* promotor. pPPαF-2myc constructs were generated in a similar manner except that they contain two c-myc-tags and the PCR products generated were digested with *Bst*Z171/*Bam*HI.

### In Vivo Pulse-Labelling

Yeast cells expressing wild-type CPY or signal sequence mutants (Table S7) were grown in YNB medium with appropriate supplements to an OD_600nm_ = 0.2, where stated cells were treated with 3 µM Fumagillin (Fluorochem) for 30 min at 30°C prior to radio-labelling. Pulse-labelling was initiated by addition of 10 µCi of [^35^S] Methionine/Cysteine mix (Perkin Elmer) per OD_600nm_ units of cells for 5 min at 30°C (20 min at 17°C for *sec61-3*). Labelling was terminated by addition of ice cold sodium azide to a final concentration of 20 mM. For each sample 5 or 10 OD_600_ units of cells were harvested.

### Denaturing Immunoprecipitation

Radiolabelled yeast cells were spheroplasted prior to addition of lysis buffer (1% SDS, 50 mM Tris-HCl, pH 7.4, and 5 mM EDTA) and then incubated at 95°C. Samples were then diluted with 5 volumes of immuno-precipitation buffer (62.5 mM Tris-HCl, pH 7.4, 1.25% (v/v) Triton-X-100, 190 mM NaCl, 6.25 mM EDTA), pre-cleared for 1 h, and then antiserum (anti-CPY or anti-αF [60],[61]) added to the supernatant. After 1 h, immune complexes were recovered with Protein A sepharose for a further hour and then washed extensively prior to elution with SDS-PAGE sample buffer. Samples were then analysed by SDS-PAGE and visualised either by phosphorimaging or autoradiography. Quantification was performed with Aida image-analyzer software (Raytek). Subsequent statistical analysis was performed using Prism 4.0 (GraphPad). Samples for scintillation counting were dissociated from the sepharose with 3% SDS for 5 min at 95°C. Dissociated protein was dried onto Whatman glass GF/A filter discs and placed in 4.5 mL of scintillant and counted in a Tricarb 2100TR liquid scintillation counter (Packard).

### In Vitro Transcription and Translation

Templates for transcription of various ppαF mRNAs were generated by PCR from plasmids pEH3 or pGF22 using appropriate primers (Table S8) and transcription carried out with SP6 polymerase. Transcriptions of OpαF mRNAs were from pGF24 or pGF25 for MR and MS OpαF, respectively, and were carried out with T7 polymerase. Translations were performed in rabbit reticulocyte lysate system (Promega) for 30 min with the inclusion of either 2.04 µCi [^35^S] Methionine or 0.04 µCi 1-[^14^C]-Acetyl Coenzyme A (Perkin Elmer) per 10 µL of reaction. Translation was terminated by addition of 2 mM cycloheximide.

Co-translational translocation of D_HC_-αF into yeast microsomes was performed using translation extracts from a strain over-expressing SRP, as described previously [42].

### Yeast Microsomes and Translocation Assays

Preparation of yeast microsomes from a *Δpep4* strain was carried out as previously described [62]. For translocation assays; 10 µL of translation reaction was incubated with 2 µL microsomes for 20 min at 30°C.

### Photocross-Linking

Wild-type and MS K5K14ppαF were translated in rabbit reticulocyte lysate as above but in the presence of ε-4-(3-trifluoro-methyldiazirino) benzoic acid (TDBA)-lysyl-tRNA and then used for photocross-linking assays as described [63]. Briefly, translations were terminated with 2 mM puromycin for 10 min at 30°C, and then treated with 0.5 mg/mL RNase A for 5 min on ice prior to depletion of ATP from the translation reaction and yeast microsomes by treatment with hexokinase/glucose. The microsomes and translation reaction were then combined, allowing targeting to occur for 15 min at 30°C. Microsomes were re-isolated by centrifugation and resuspended in membrane storage buffer. Samples were irradiated with uv light (365 nm, 15 mW/cm^2^) twice for 5 s and then precipitated with ethanol and analysed directly or following denaturing immuno-precipitation with Sec61 antiserum [64].

### N-Terminal Biotinylation

In vitro translations (20 µL scale), programmed with lysine-free OpαF mRNAs, were performed as above in the presence of [^35^S] methionine. Proteins were sequentially precipitated with ammonium sulphate, then ethanol. The samples were then denatured in PBS+1% SDS for 10 min at 65°C. Free N-termini were modified by treatment with 1 mM sulpho-NHS-SS-Biotin (Pierce) for 20 min at 37°C. After removal of free biotinylation reagent by acetone precipitation, samples were resuspended in PBS+0.1% SDS and then biotinylated proteins recovered on immobilized-streptavidin beads (Pierce). Beads were washed 5 times with PBS+0.1% SDS and bound protein eluted in SDS-PAGE sample buffer.

## Supporting Information

Figure S1 Quantification of CPY translocation in the presence and absence of MetAP activity. Pulse-labelling of WT (MK) CPY and mutants with A, C, E, G, and S inserted at P2 was performed in wild-type (*MAP1 Δprc1 Δpep4*) and Δ*map1* (*Δprc1 Δpep4*) yeast cells in the presence and absence of the Map2 inhibitor fumagillin. CPY was immunoprecipitated and analysed by SDS-PAGE and phosphorimaging (see Figure 2). Translocation efficiency was determined from quantification of the relative amounts of glycosylated-CPY and non-translocated pCPY. The data are displayed graphically and represent the means of three independent experiments. Error bars represent the standard error of the mean. Asterisks represent statistically significant differences to the untreated wild-type (*MAP1*) strain with *p*<0.01 (**) and *p*<0.001 (***) according to the two-way analysis of variance.
(TIF)

Figure S2 MS-pPdi1p is Methionine-cleaved and N-acetylated in vivo. MS-pPdi1p-myc was affinity purified from yeast cells with anti-myc antiserum and analysed by SDS-PAGE and staining with Coomassie brilliant blue (Text S1). The MS-pPdi1p-myc precursor band was excised, digested with elastase, and analysed by LC-MS/MS (Text S1). Product ion spectra and associated fragmentation tables, which list all the fragment ions observed (highlighted), are shown for two N-terminal peptides. No peptides corresponding to an unmodified N-terminus were detected in the analysis.
(TIF)

Figure S3 N-acetylation of ppαF blocks translocation in vitro. Wild-type (MR), MSR, and MSRR ppαF were translated in vitro in rabbit reticulocyte lysate and then incubated with yeast microsomes (yRM). Position of non-translocated (ppαF) and signal-sequence cleaved, glycosylated (g-pαF) are indicated. (*) Ubiquitinylated ppαF generated in the absence of microsomes.
(TIF)

Figure S4 D_HC_-αF translocation is insensitive to a P2 residue that can promote N-acetylation. (A) D_HC_-αF comprises ppαF with the hydrophobic core of the signal sequence replaced with that of DPAP B, creating an SRP-dependent substrate. D_HC_-αF with the endogenous P2 residue (MR) or with a serine inserted at position 2 (MS) were translated in vitro in a yeast extract supplemented with [^35^S] methionine in the presence or absence of yeast microsomes (yRM). Translated proteins were immunoprecipitated with anti-αF antibodies prior to analysis by SDS-PAGE and phosphorimaging. Positions of the unprocessed (D_HC_αF) and glycosylated (g-D_HC_αF) forms of the protein are indicated. (B) WT and MS ppαF were translated in yeast extract in the presence of [^35^S] methionine and incubated with or without yeast microsomes.
(TIF)

Figure S5 Peak hydrophobicity analysis of Yeast Signal Sequences. Mean peak hydrophobicity of yeast signal sequences group according to their predicted N-terminal processing. Peak hydrophobicity determined based on Kyte-Doolittle [57] with a window size of 11. The “acetylated,” “methionine cleaved not acetylated,” and “non-processed” groups had mean peak hydrophobicities of 2.593±0.0657 (SEM), 2.518±0.0673, and 2.333±0.0352, respectively. The “acetylated” and “cleaved not acetylated” groups differed significantly from the “unprocessed” group (*p*<0.01 and *p*<0.05, respectively, one-way ANOVA with Tukey's multiple comparison test). The acetylated and cleaved group were not significantly different. Note that only two signal sequences of the acetylated group (<6%) had a peak hydrophobicity of less than 2, the threshold for interaction with SRP [30].
(TIF)

Table S1 N-terminal sequence and predicted processing of yeast signal sequences.
(PDF)

Table S2 N-terminal sequence and predicted processing of cytosolic proteins.
(PDF)

Table S3 Relative amino acid frequency at position 2 by compartment in yeast.
(PDF)

Table S4 Predicted relative frequency of N-terminal methionine cleavage.
(PDF)

Table S5 Relative P2 frequency of signal sequences from different organisms.
(PDF)

Table S6 Predicted frequency of N-terminal processing of signal sequences from different organisms.
(PDF)

Table S7 Yeast strains used in this study.
(PDF)

Table S8 Oligonucleotides used in this study.
(PDF)

Table S9 Plasmids used in this study.
(PDF)

Text S1 Supporting methods.
(PDF)

## Abbreviations

CPY: carboxypeptidase Y
D_HC_-αF: prepro alpha mating factor with the hydrophobic core of the signal sequence replaced with that of DPAP B
DPAP B: dipeptidyl aminopeptidase B
ER: endoplasmic reticulum
MetAP: methionine aminopeptidase
NAT: N-α-acetyl transferase
opαF: Ost1 signal sequence fused to pro-alpha mating factor
OPY: Ost1 signal sequence fused to mature region of CPY
P2: amino acid residue 2
ppαF: prepro-alpha mating factor
ppCPY: prepro-CPY
SRP: signal recognition particle
TDBA: 4-(3-trifluoro-methyldiazirino) benzoic acid
