# Supplementary figures and images for: N-Terminal Acetylation Inhibits Protein Targeting to the Endoplasmic Reticulum
Source: PLoS Biol. 2011 May 31;9(5):e1001073. doi: 10.1371/journal.pbio.1001073 (PMC3104963; doi:10.1371/journal.pbio.1001073)

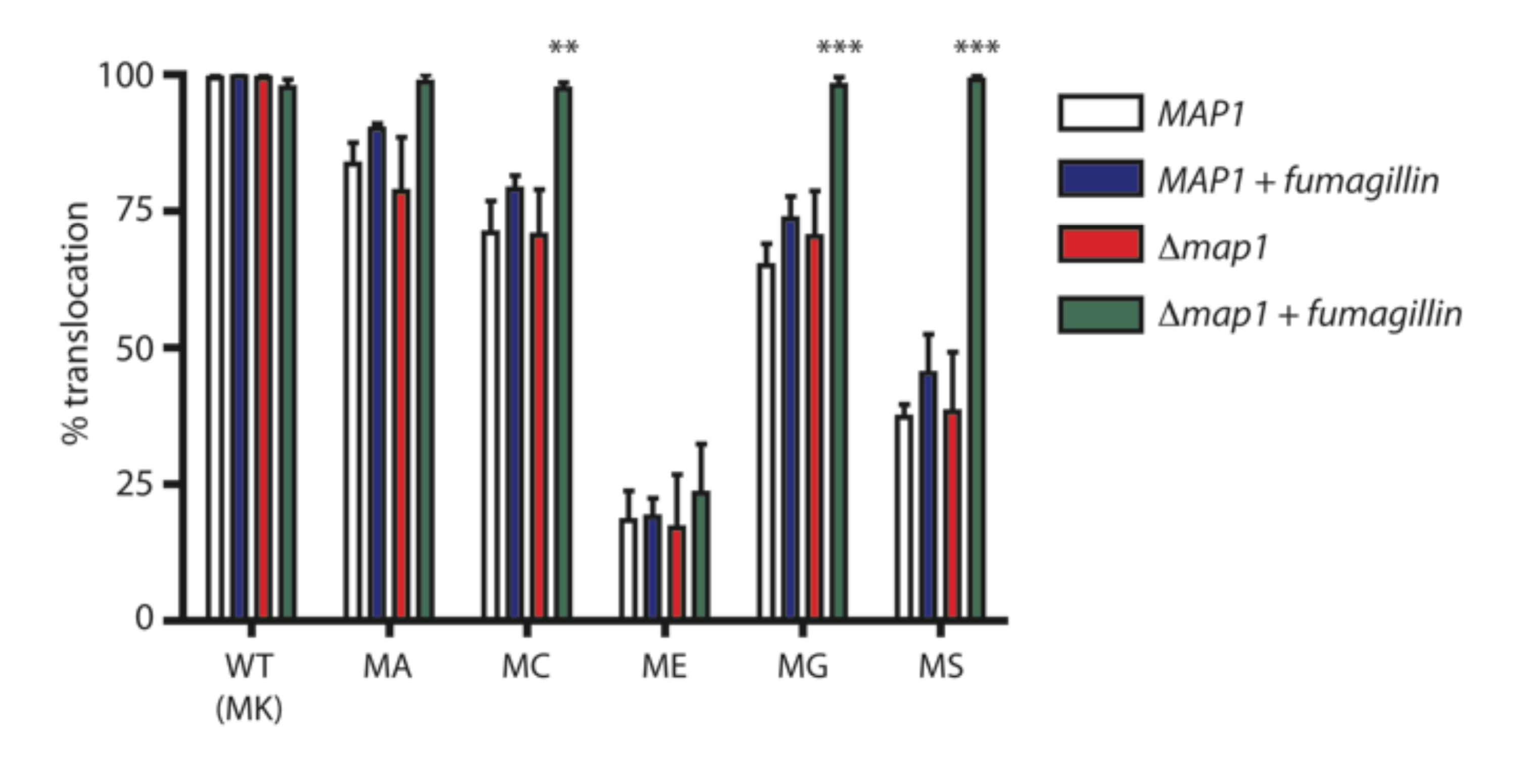

Supplement: Figure S1 — Quantification of CPY translocation in the presence and absence of MetAP activity. Pulse-labelling of WT (MK) CPY and mutants with A, C, E, G, and S inserted at P2 was performed in wild-type (MAP1 Δprc1 Δpep4) and Δmap1 (Δprc1 Δpep4) yeast cells in the presence and absence of the Map2 inhibitor fumagillin. CPY was immunoprecipitated and analysed by SDS-PAGE and phosphorimaging (see Figure 2). Translocation efficiency was determined from quantification of the relative amounts of glycosylated-CPY and non-translocated pCPY. The data are displayed graphically and represent the means of three independent experiments. Error bars represent the standard error of the mean. Asterisks represent statistically significant differences to the untreated wild-type (MAP1) strain with p<0.01 (**) and p<0.001 (***) according to the two-way analysis of variance. (TIF) [file pbio.1001073.s001.tif]

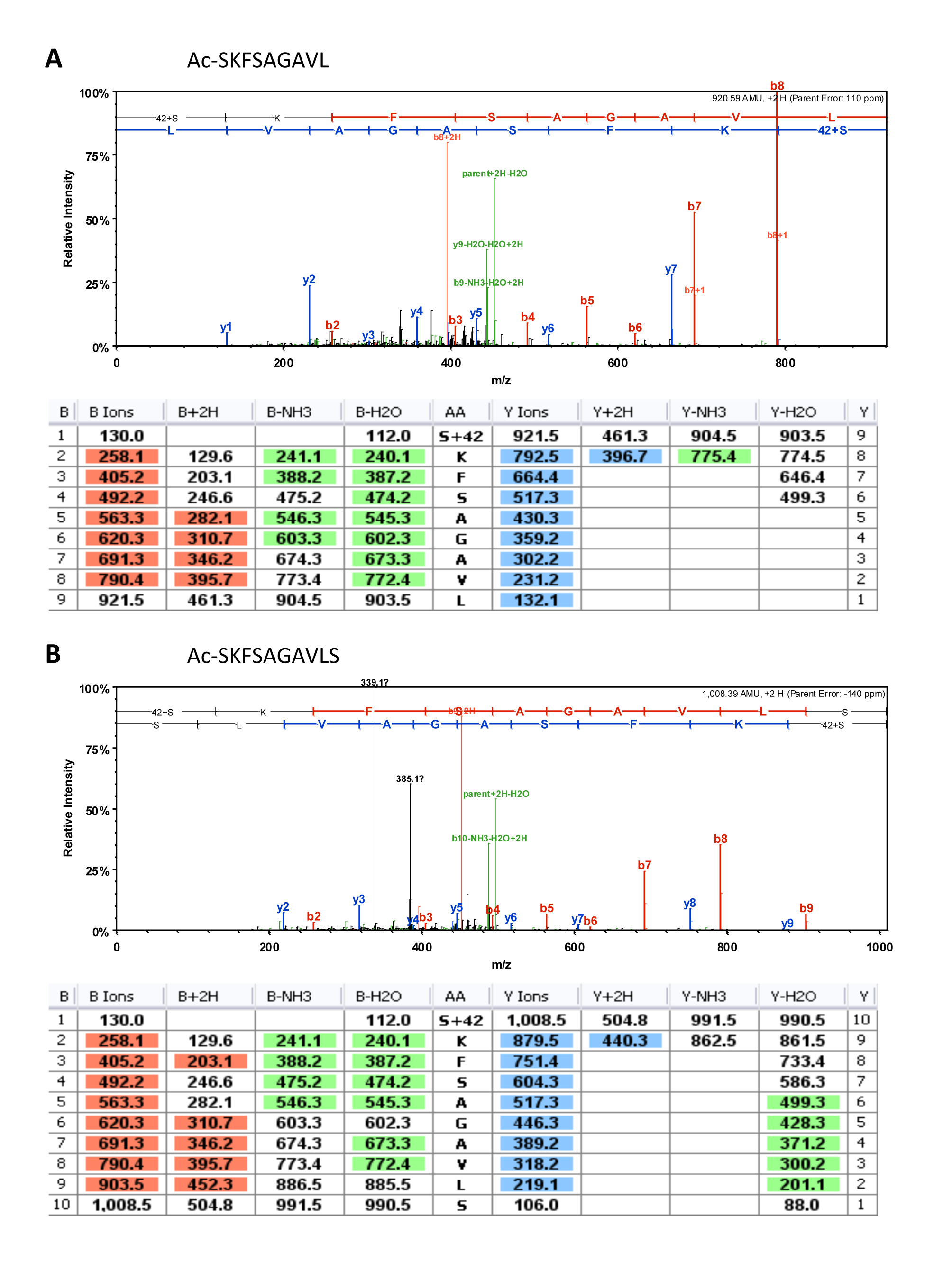

Supplement: Figure S2 — MS-pPdi1p is Methionine-cleaved and N-acetylated in vivo. MS-pPdi1p-myc was affinity purified from yeast cells with anti-myc antiserum and analysed by SDS-PAGE and staining with Coomassie brilliant blue (Text S1). The MS-pPdi1p-myc precursor band was excised, digested with elastase, and analysed by LC-MS/MS (Text S1). Product ion spectra and associated fragmentation tables, which list all the fragment ions observed (highlighted), are shown for two N-terminal peptides. No peptides corresponding to an unmodified N-terminus were detected in the analysis. (TIF) [file pbio.1001073.s002.tif]

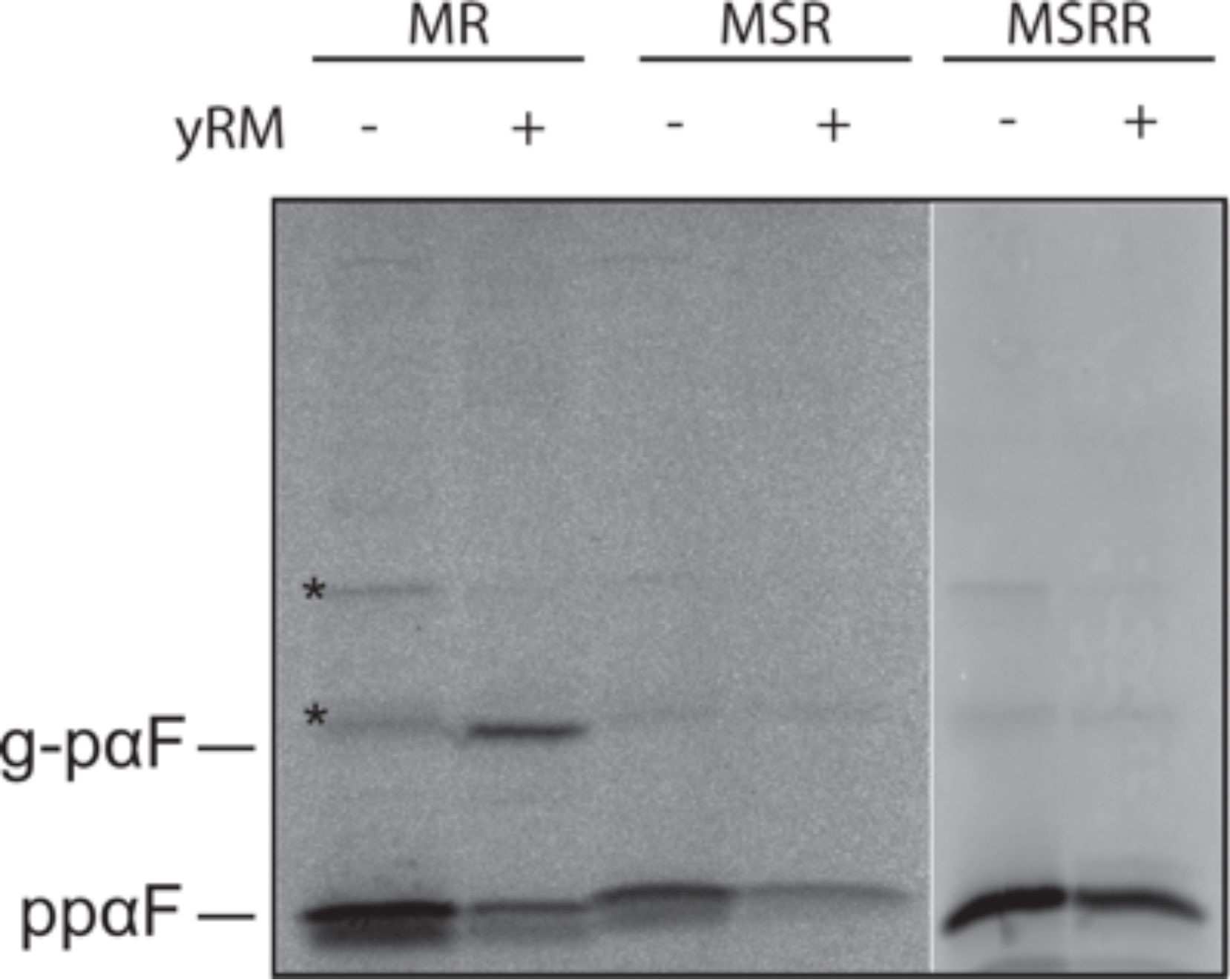

Supplement: Figure S3 — N-acetylation of ppαF blocks translocation in vitro. Wild-type (MR), MSR, and MSRR ppαF were translated in vitro in rabbit reticulocyte lysate and then incubated with yeast microsomes (yRM). Position of non-translocated (ppαF) and signal-sequence cleaved, glycosylated (g-pαF) are indicated. (*) Ubiquitinylated ppαF generated in the absence of microsomes. (TIF) [file pbio.1001073.s003.tif]

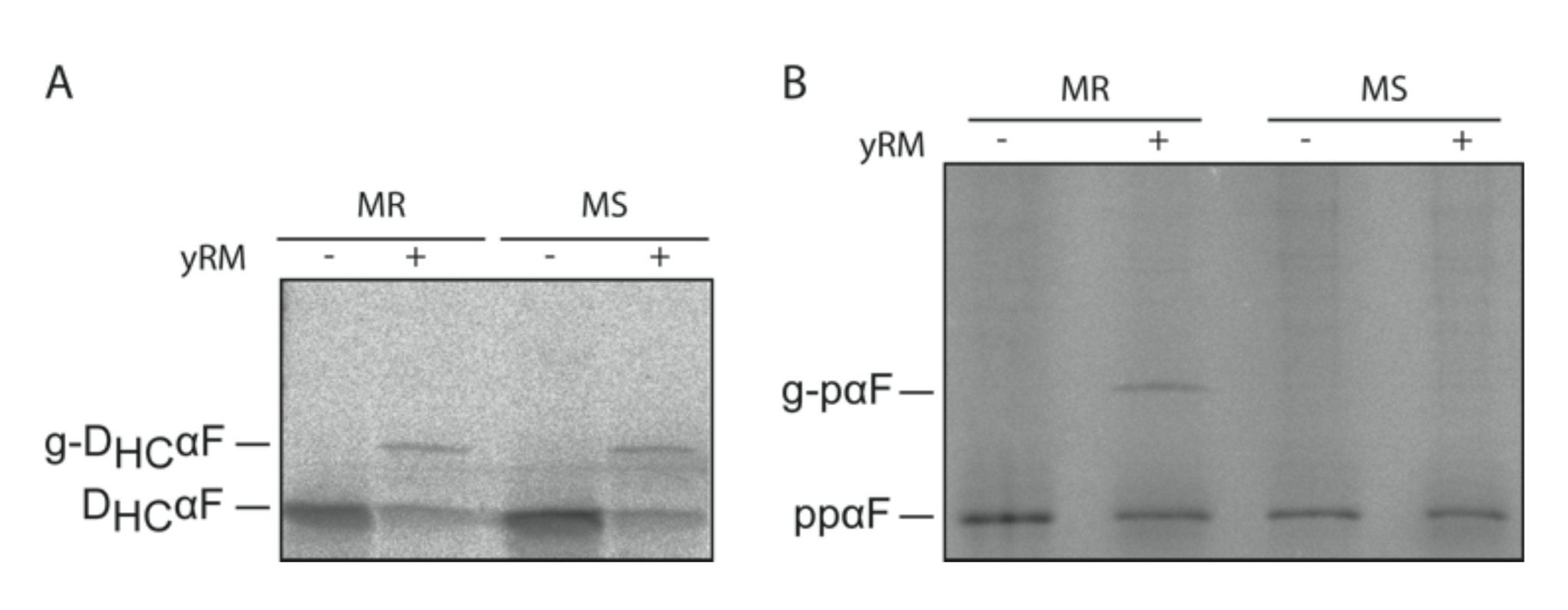

Supplement: Figure S4 — DHC-αF translocation is insensitive to a P2 residue that can promote N-acetylation. (A) DHC-αF comprises ppαF with the hydrophobic core of the signal sequence replaced with that of DPAP B, creating an SRP-dependent substrate. DHC-αF with the endogenous P2 residue (MR) or with a serine inserted at position 2 (MS) were translated in vitro in a yeast extract supplemented with [35S] methionine in the presence or absence of yeast microsomes (yRM). Translated proteins were immunoprecipitated with anti-αF antibodies prior to analysis by SDS-PAGE and phosphorimaging. Positions of the unprocessed (DHCαF) and glycosylated (g-DHCαF) forms of the protein are indicated. (B) WT and MS ppαF were translated in yeast extract in the presence of [35S] methionine and incubated with or without yeast microsomes. (TIF) [file pbio.1001073.s004.tif]

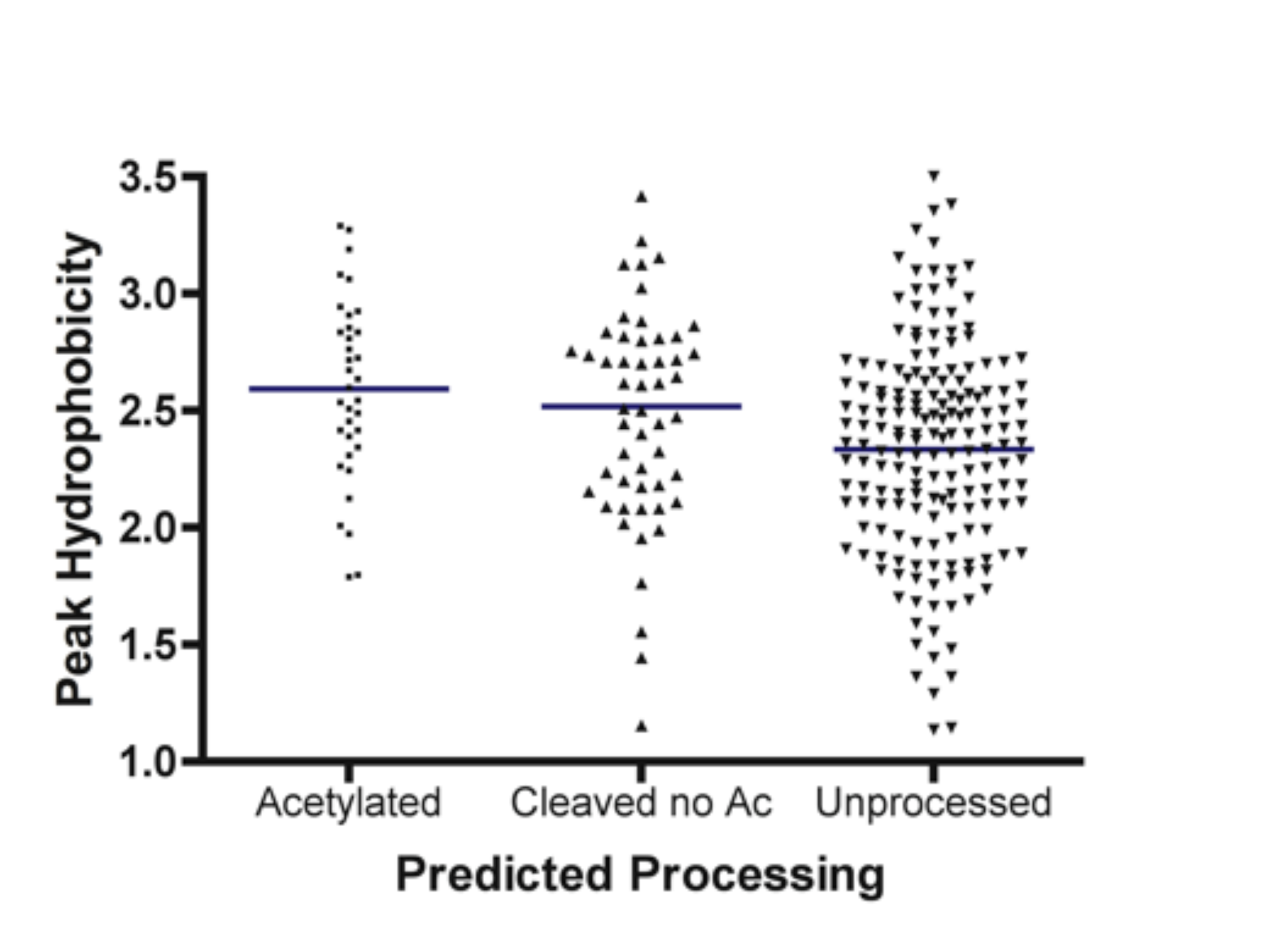

Supplement: Figure S5 — Peak hydrophobicity analysis of Yeast Signal Sequences. Mean peak hydrophobicity of yeast signal sequences group according to their predicted N-terminal processing. Peak hydrophobicity determined based on Kyte-Doolittle [57] with a window size of 11. The “acetylated,” “methionine cleaved not acetylated,” and “non-processed” groups had mean peak hydrophobicities of 2.593±0.0657 (SEM), 2.518±0.0673, and 2.333±0.0352, respectively. The “acetylated” and “cleaved not acetylated” groups differed significantly from the “unprocessed” group (p<0.01 and p<0.05, respectively, one-way ANOVA with Tukey's multiple comparison test). The acetylated and cleaved group were not significantly different. Note that only two signal sequences of the acetylated group (<6%) had a peak hydrophobicity of less than 2, the threshold for interaction with SRP [30]. (TIF) [file pbio.1001073.s005.tif]
