# Supplementary material for: N-Terminal Acetylation Inhibits Protein Targeting to the Endoplasmic Reticulum
Source: PLoS Biol. 2011 May 31;9(5):e1001073. doi: 10.1371/journal.pbio.1001073 (PMC3104963; doi:10.1371/journal.pbio.1001073)
Supplement: Table S1 — N-terminal sequence and predicted processing of yeast signal sequences. (PDF) [file pbio.1001073.s006.pdf]

Table S1 N-terminal sequence and predicted processing of yeast signal sequences

Protein	N-terminal residues	Predicted Met cleavage	Predicted Acetylation*
ADP1	MG	Cleaved	Non-acetylated
AGA1	MT	Cleaved	Non-acetylated
AGA2	MQ	Uncleaved	Non-acetylated
AIM6	MLG	Uncleaved	Non-acetylated
ANS1	MK	Uncleaved	Non-acetylated
APE3	MHF	Uncleaved	Non-acetylated
ASP3-1	MR	Uncleaved	Non-acetylated
ASP3-2	MR	Uncleaved	Non-acetylated
ASP3-3	MR	Uncleaved	Non-acetylated
ASP3-4	MR	Uncleaved	Non-acetylated
ATG15	MLG	Uncleaved	Non-acetylated
ATG27	MV	Cleaved	Non-acetylated
ATH1	MK	Uncleaved	Non-acetylated
AWA1	MFN	Uncleaved	Acetylated
AXL2	MT	Cleaved	Non-acetylated
BAR1	MS	Cleaved	Acetylated
BGL2	MR	Uncleaved	Non-acetylated
BIG1	MQ	Uncleaved	Non-acetylated
CCW12	MQ	Uncleaved	Non-acetylated
CCW14	MR	Uncleaved	Non-acetylated
CDA1	MK	Uncleaved	Non-acetylated
CDA2	MR	Uncleaved	Non-acetylated
CIS3	MQ	Uncleaved	Non-acetylated
CNE1	MK	Uncleaved	Non-acetylated
CPR4	MWL	Uncleaved	Non-acetylated
CPR5	MK	Uncleaved	Non-acetylated
CRH1	MK	Uncleaved	Non-acetylated
CRR1	MR	Uncleaved	Non-acetylated
CSG2	MS	Cleaved	Acetylated
CTS1	MS	Cleaved	Acetylated
CTS2	MV	Cleaved	Non-acetylated
CWH43	MLI	Uncleaved	Non-acetylated
CWP1	MK	Uncleaved	Non-acetylated
CWP2	MQ	Uncleaved	Non-acetylated
CYP2	MK	Uncleaved	Non-acetylated
DAN1	MS	Cleaved	Acetylated
DAN2	MV	Cleaved	Non-acetylated
DAN3	MV	Cleaved	Non-acetylated
DAN4	MV	Cleaved	Non-acetylated
DCW1	MLV	Uncleaved	Non-acetylated
DDR2	MK	Uncleaved	Non-acetylated
DFG5	MI	Uncleaved	Non-acetylated
DIA3	MV	Cleaved	Non-acetylated
DSE2	MK	Uncleaved	Non-acetylated
DSE4	MQ	Uncleaved	Non-acetylated
ECM14	MLH	Uncleaved	Non-acetylated
ECM33	MQ	Uncleaved	Non-acetylated
EGT2	MN	Uncleaved	Acetylated *

EMC1	MK	Uncleaved	Non-acetylated
EMP24	MAS	Cleaved	Acetylated
EMP46	MT	Cleaved	Non-acetylated
EMP47	MMM	Uncleaved	Non-acetylated
EMP70	MI	Uncleaved	Non-acetylated
EPS1	MK	Uncleaved	Non-acetylated
ERJ5	MN	Uncleaved	Acetylated *
ERO1	MR	Uncleaved	Non-acetylated
ERP1	MLL	Uncleaved	Non-acetylated
ERP2	MI	Uncleaved	Non-acetylated
ERP3	MS	Cleaved	Acetylated
ERP4	MR	Uncleaved	Non-acetylated
ERP5	MR	Uncleaved	Non-acetylated
ERP6	MLS	Uncleaved	Non-acetylated
ERV25	MQ	Uncleaved	Non-acetylated
EUG1	MQ	Uncleaved	Non-acetylated
EXG1	MLS	Uncleaved	Non-acetylated
EXG2	MP	Cleaved	Non-acetylated
FET3	MT	Cleaved	Non-acetylated
FET5	MLF	Uncleaved	Non-acetylated
FIG2	MN	Uncleaved	Acetylated *
FIT1	MK	Uncleaved	Non-acetylated
FIT2	MK	Uncleaved	Non-acetylated
FIT3	MK	Uncleaved	Non-acetylated
FLC1	MQ	Uncleaved	Non-acetylated
FLC2	MI	Uncleaved	Non-acetylated
FLC3	MR	Uncleaved	Non-acetylated
FLO1	MT	Cleaved	Non-acetylated
FLO10	MP	Cleaved	Non-acetylated
FLO11	MQ	Uncleaved	Non-acetylated
FLO5	MT	Cleaved	Non-acetylated
FLO9	MS	Cleaved	Acetylated
FMN1	MFT	Uncleaved	Non-acetylated
FPR2	MMF	Uncleaved	Non-acetylated
FRE1	MV	Cleaved	Non-acetylated
FRE2	MHW	Uncleaved	Non-acetylated
FRE3	MY	Uncleaved	Non-acetylated
FRE4	MLL	Uncleaved	Non-acetylated
FRE5	MLF	Uncleaved	Non-acetylated
FRE6	MHR	Uncleaved	Acetylated
GAB1	MD	Uncleaved	Acetylated
GAS1	MLF	Uncleaved	Non-acetylated
GAS2	MN	Uncleaved	Acetylated *
GAS3	MQ	Uncleaved	Non-acetylated
GAS4	MMV	Uncleaved	Non-acetylated
GAS5	MLL	Uncleaved	Non-acetylated
GPI16	MI	Uncleaved	Non-acetylated
GPI17	MS	Cleaved	Acetylated
GPI8	MR	Uncleaved	Non-acetylated
GRX6	MI	Uncleaved	Non-acetylated
GTB1	MV	Cleaved	Non-acetylated
HKR1	MV	Cleaved	Non-acetylated
HOR7	MK	Uncleaved	Non-acetylated

HPF1	MFN	Uncleaved	Acetylated
HRD3	MI	Uncleaved	Non-acetylated
HSP150	MQ	Uncleaved	Non-acetylated
IRC22	MR	Uncleaved	Non-acetylated
IRE1	MR	Uncleaved	Non-acetylated
JEM1	MI	Uncleaved	Non-acetylated
KAR2	MFF	Uncleaved	Non-acetylated
KAR5	MFE	Uncleaved	Non-acetylated
KEX1	MFY	Uncleaved	Non-acetylated
KEX2	MK	Uncleaved	Non-acetylated
KNH1	MLI	Uncleaved	Non-acetylated
KRE1	MMR	Uncleaved	Acetylated
KRE27	MS	Cleaved	Acetylated
KRE5	MR	Uncleaved	Non-acetylated
KRE9	MR	Uncleaved	Non-acetylated
LHS1	MR	Uncleaved	Non-acetylated
LRC1	MLG	Uncleaved	Non-acetylated
MEL1	MFA	Uncleaved	Acetylated
MEL2	MFA	Uncleaved	Acetylated
MEL5	MFA	Uncleaved	Acetylated
MEL6	MFA	Uncleaved	Acetylated
MFALPHA	MR	Uncleaved	Non-acetylated
MFALPHA2	MK	Uncleaved	Non-acetylated
MID1	MI	Uncleaved	Non-acetylated
MID2	MLS	Uncleaved	Non-acetylated
MKC7	MK	Uncleaved	Non-acetylated
MNL1	MV	Cleaved	Non-acetylated
MNN5	MLI	Uncleaved	Non-acetylated
MPD1	MLF	Uncleaved	Non-acetylated
MPD2	MK	Uncleaved	Non-acetylated
MRL1	MLK	Uncleaved	Acetylated
MSB2	MQ	Uncleaved	Non-acetylated
MTL1	MAS	Cleaved	Acetylated
MUC1	MQ	Uncleaved	Non-acetylated
NCP1	MP	Cleaved	Non-acetylated
NCR1	MN	Uncleaved	Acetylated *
NPC2	MT	Cleaved	Acetylated
NVJ1	MT	Cleaved	Acetylated
OST1	MR	Uncleaved	Non-acetylated
OST3	MN	Uncleaved	Acetylated *
OST6	MK	Uncleaved	Non-acetylated
PAU10	MV	Cleaved	Non-acetylated
PAU13	MV	Cleaved	Non-acetylated
PAU15	MV	Cleaved	Non-acetylated
PDI1	MK	Uncleaved	Non-acetylated
PEP1	MI	Uncleaved	Non-acetylated
PEP4	MFS	Uncleaved	Non-acetylated
PER1	MR	Uncleaved	Non-acetylated
PGA1	MV	Cleaved	Non-acetylated
PGU1	MI	Uncleaved	Non-acetylated
PHO11	MLK	Uncleaved	Acetylated
PHO12	MLK	Uncleaved	Acetylated
PHO3	MFK	Uncleaved	Acetylated

PHO5	MFK	Uncleaved	Acetylated
PIR1	MQ	Uncleaved	Non-acetylated
PIR3	MQ	Uncleaved	Non-acetylated
PLB1	MK	Uncleaved	Non-acetylated
PLB2	MQ	Uncleaved	Non-acetylated
PLB3	MI	Uncleaved	Non-acetylated
PRB1	MK	Uncleaved	Non-acetylated
PRC1	MK	Uncleaved	Non-acetylated
PRY1	MK	Uncleaved	Non-acetylated
PRY2	MK	Uncleaved	Non-acetylated
PRY3	MLE	Uncleaved	Non-acetylated
PST1	MQ	Uncleaved	Non-acetylated
PST2	MP	Cleaved	Non-acetylated
PTM1	MR	Uncleaved	Non-acetylated
RAX2	MFV	Uncleaved	Non-acetylated
RNY1	MLL	Uncleaved	Non-acetylated
ROT1	MWS	Uncleaved	Non-acetylated
ROT2	MV	Cleaved	Non-acetylated
RRT12	MK	Uncleaved	Non-acetylated
SAG1	MFT	Uncleaved	Non-acetylated
SCJ1	MI	Uncleaved	Non-acetylated
SCW10	MR	Uncleaved	Non-acetylated
SCW11	MI	Uncleaved	Non-acetylated
SCW4	MR	Uncleaved	Non-acetylated
SED1	MK	Uncleaved	Non-acetylated
SHE10	MG	Cleaved	Non-acetylated
SIA1	MR	Uncleaved	Non-acetylated
SIL1	MV	Cleaved	Non-acetylated
SIM1	MK	Uncleaved	Non-acetylated
SLG1	MR	Uncleaved	Non-acetylated
SLP1	MAN	Cleaved	Non-acetylated
SOP4	MFS	Uncleaved	Non-acetylated
SPI1	MLS	Uncleaved	Non-acetylated
SPO19	MK	Uncleaved	Non-acetylated
SPR1	MV	Cleaved	Non-acetylated
SPS100	MK	Uncleaved	Non-acetylated
SPS22	MN	Uncleaved	Acetylated *
SRL1	MLQ	Uncleaved	Non-acetylated
SSP120	MR	Uncleaved	Non-acetylated
STA1	MV	Cleaved	Non-acetylated
STA2	MQ	Uncleaved	Non-acetylated
SUC1	ML	Uncleaved	Non-acetylated
SUC2	MLL	Uncleaved	Non-acetylated
SUC4	ML	Uncleaved	Non-acetylated
SUN4	MK	Uncleaved	Non-acetylated
SVS1	MI	Uncleaved	Non-acetylated
SWP1	MQ	Uncleaved	Non-acetylated
TED1	MLR	Uncleaved	Acetylated
THI22	MV	Cleaved	Non-acetylated
TIP1	MS	Cleaved	Acetylated
TIR1	MAY	Cleaved	Non-acetylated
TIR2	MAY	Cleaved	Non-acetylated
TIR3	MS	Cleaved	Acetylated

TIR4	MAY	Cleaved	Non-acetylated
TMN2	MK	Uncleaved	Non-acetylated
TMN3	MR	Uncleaved	Non-acetylated
TOS1	MLQ	Uncleaved	Non-acetylated
UIP5	MS	Cleaved	Acetylated
UTR2	MAI	Cleaved	Non-acetylated
VOA1	MV	Cleaved	Non-acetylated
VTH1	MAL	Cleaved	Non-acetylated
WBP1	MR	Uncleaved	Non-acetylated
WSC2	MHL	Uncleaved	Non-acetylated
WSC3	ME	Uncleaved	Acetylated
WSC4	MQ	Uncleaved	Non-acetylated
YBL008W-A	MK	Uncleaved	Non-acetylated
YBR013C	MI	Uncleaved	Non-acetylated
YBR200W-A	MLL	Uncleaved	Non-acetylated
YCL012C	MK	Uncleaved	Non-acetylated
YCL048W-A	MQ	Uncleaved	Non-acetylated
YCL049C	MFS	Uncleaved	Non-acetylated
YCR012C	MK	Uncleaved	Non-acetylated
YDR053W	MR	Uncleaved	Non-acetylated
YDR134C	MQ	Uncleaved	Non-acetylated
YDR246W-A	MR	Uncleaved	Non-acetylated
YDR262W	MI	Uncleaved	Non-acetylated
YDR366C	MV	Cleaved	Non-acetylated
YDR415C	MR	Uncleaved	Non-acetylated
YDR524C-B	MQ	Uncleaved	Non-acetylated
YER067W	MT	Cleaved	Non-acetylated
YFL051C	MS	Cleaved	Acetylated
YFR012W-A	MLP	Uncleaved	Non-acetylated
YGP1	MK	Uncleaved	Non-acetylated
YGR079W	MS	Cleaved	Acetylated
YHC3	MS	Cleaved	Acetylated
YHL017W	MD	Uncleaved	Acetylated
YHL042W	MK	Uncleaved	Non-acetylated
YHR138C	MK	Uncleaved	Non-acetylated
YHR214W	MFN	Uncleaved	Acetylated
YIL156W-B	MT	Cleaved	Non-acetylated
YIL169C	MFN	Uncleaved	Acetylated
YJL052C	MHL	Uncleaved	Non-acetylated
YJL160c	MHY	Uncleaved	Non-acetylated
YJL171C	MLQ	Uncleaved	Non-acetylated
YJR120W	MR	Uncleaved	Non-acetylated
YKL018C-A	MLG	Uncleaved	Non-acetylated
YLR001C	MN	Uncleaved	Acetylated *
YLR040c	MI	Uncleaved	Non-acetylated
YLR042C	MK	Uncleaved	Non-acetylated
YLR104W	MS	Cleaved	Acetylated
YLR194C	MK	Uncleaved	Non-acetylated
YLR406C-A	MI	Uncleaved	Non-acetylated
YLR413W	MN	Uncleaved	Acetylated *
YLR414C	MR	Uncleaved	Non-acetylated
YMR247W-A	MAH	Cleaved	Non-acetylated
YMR272W-B	MR	Uncleaved	Non-acetylated

YMR315W-A	MT	Cleaved	Non-Acetylated
YNL019C	MLY	Uncleaved	Non-acetylated
YNL024C-A	MS	Cleaved	Acetylated
YNL033W	MLY	Uncleaved	Non-acetylated
YNL217W	ME	Uncleaved	Acetylated
YOR008C-A	MWR	Uncleaved	Acetylated
YOR214C	MLG	Uncleaved	Non-acetylated
YOR365C	MLP	Uncleaved	Non-acetylated
YOR389W	MR	Uncleaved	Non-acetylated
YOS9	MQ	Uncleaved	Non-acetylated
YPS1	MK	Uncleaved	Non-acetylated
YPS3	MK	Uncleaved	Non-acetylated
YPS5	MQ	Uncleaved	Non-acetylated
YPS6	MQ	Uncleaved	Non-acetylated
YPS7	MT	Cleaved	Non-acetylated
YSP3	MK	Uncleaved	Non-acetylated
ZPS1	MK	Uncleaved	Non-acetylated

* MN acetylation is predicted in ony 55% of cases
