# Supplementary material for: N-Terminal Acetylation Inhibits Protein Targeting to the Endoplasmic Reticulum
Source: PLoS Biol. 2011 May 31;9(5):e1001073. doi: 10.1371/journal.pbio.1001073 (PMC3104963; doi:10.1371/journal.pbio.1001073)
Supplement: Table S2 — N-terminal sequence and predicted processing of cytosolic proteins. (PDF) [file pbio.1001073.s007.pdf]

Table S2 N-terminal sequence and predicted processing of yeast cytosolic proteins

Protein	N-terminal residues	Predicted Met cleavage	Predicted Acetylation*
AAT2	MS	cleaved	Acetylated
ABZ2	MS	cleaved	Acetylated
ACS2	MT	cleaved	non-acetylated
ACT1	MD	uncleaved	Acetylated
ADE1	MS	cleaved	Acetylated
ADE2	MD	uncleaved	Acetylated
ADE3	MAG	cleaved	non-acetylated
ADK1	MS	cleaved	Acetylated
ALD6	MT	cleaved	non-acetylated
ALD6	MT	cleaved	non-acetylated
ANB1	MS	cleaved	Acetylated
APL3	MD	uncleaved	Acetylated
APL6	MV	cleaved	non-acetylated
APS3	MI	uncleaved	non-acetylated
ARD1	MP	cleaved	non-acetylated
ARF1	MG	cleaved	non-acetylated
ARG4	MS	cleaved	Acetylated
ARK1	MN	uncleaved	Acetylated *
ARP14	MS	cleaved	Acetylated
ARP2	MD	uncleaved	Acetylated
ASC1	MAS	cleaved	Acetylated
ASP1	MK	uncleaved	non-acetylated
ATG1	MG	cleaved	non-acetylated
ATG11	MAD	cleaved	non-acetylated
ATS1	MS	cleaved	Acetylated
ATX1	MAE	cleaved	Acetylated
AVO2	MLK	uncleaved	Acetylated
BAT2	MT	cleaved	non-acetylated
BAT2	MT	cleaved	non-acetylated
BCY1	MV	cleaved	non-acetylated
BEM1	MLK	uncleaved	Acetylated
BIM1	MS	cleaved	Acetylated
BMH1	MS	cleaved	Acetylated
BMH2	MS	cleaved	Acetylated
BNI1	MLK	uncleaved	Acetylated
BTS1	ME	uncleaved	Acetylated
BTT1	MP	cleaved	non-acetylated
CCR4	MN	uncleaved	Acetylated *
CCT3	MQ	uncleaved	non-acetylated
CCT4	MS	cleaved	Acetylated
CCT6	MS	cleaved	Acetylated
CCT7	MN	uncleaved	Acetylated *
CDC14	MR	uncleaved	non-acetylated
CDC19	MS	cleaved	Acetylated
CDC42	MQ	uncleaved	non-acetylated
CDC48	MG	cleaved	non-acetylated
CDC60	MS	cleaved	Acetylated
CDC8	MMG	uncleaved	non-acetylated
CIA1	MAS	cleaved	Acetylated

CKA2	MP	cleaved	non-acetylated
CKB2	MG	cleaved	non-acetylated
CLA4	MS	cleaved	Acetylated
CMD1	MS	cleaved	Acetylated
CMK1	MD	uncleaved	Acetylated
CPR1	MS	cleaved	Acetylated
CSL4	MAC	cleaved	non-acetylated
CYS3	MT	cleaved	non-acetylated
DCC1	MS	cleaved	Acetylated
DCP2	MS	cleaved	Acetylated
DDP1	MG	cleaved	non-acetylated
DED81	MS	cleaved	Acetylated
DIA2	MS	cleaved	Acetylated
DOA1	MG	cleaved	non-acetylated
DOG2	MP	cleaved	non-acetylated
DYS1	MS	cleaved	Acetylated
DYS1	MS	cleaved	Acetylated
EBS1	ME	uncleaved	Acetylated
EDE1	MAS	cleaved	Acetylated
EFB1	MAS	cleaved	Acetylated
EGD1	MP	cleaved	non-acetylated
EGD2	MS	cleaved	Acetylated
ERG10	MS	cleaved	Acetylated
ERG8	MS	cleaved	Acetylated
EXO70	MP	cleaved	non-acetylated
EXO84	MV	cleaved	non-acetylated
FAB1	MS	cleaved	Acetylated
FAS1	MD	uncleaved	Acetylated
FAS2	MK	uncleaved	non-acetylated
FBA1	MG	cleaved	non-acetylated
FES1	ME	uncleaved	Acetylated
FOL2	MHN	uncleaved	Acetylated
FPR1	MS	cleaved	Acetylated
FRS1	MP	cleaved	non-acetylated
FUN11	MS	cleaved	Acetylated
GAL1	MT	cleaved	non-acetylated
GAL10	MT	cleaved	non-acetylated
GCD11	MS	cleaved	Acetylated
GCN2	MS	cleaved	Acetylated
GET3	MD	uncleaved	Acetylated
GGA2	MS	cleaved	Acetylated
GIM3	ME	uncleaved	Acetylated
GIM4	ME	uncleaved	Acetylated
GLK1	MS	cleaved	Acetylated
GTO3	MS	cleaved	Acetylated
GUA1	MAA	cleaved	non-acetylated
GUS1	MP	cleaved	non-acetylated
HAC1	ME	uncleaved	Acetylated
HCH1	MV	cleaved	non-acetylated
HEK2	MS	cleaved	Acetylated
HIS3	MT	cleaved	non-acetylated
HIS4	MV	cleaved	non-acetylated
HOM2	MAG	cleaved	non-acetylated

HOM3	MP	cleaved	non-acetylated
HOM6	MS	cleaved	Acetylated
HTS1	MLS	uncleaved	non-acetylated
HUL5	MLN	uncleaved	Acetylated
HXK1	MV	cleaved	non-acetylated
HXK2	MV	cleaved	non-acetylated
HYP2	MS	cleaved	Acetylated
IDP2	MT	cleaved	non-acetylated
ILS1	MS	cleaved	Acetylated
IPP1	MT	cleaved	non-acetylated
IRA2	MS	cleaved	Acetylated
JSN1	MD	uncleaved	Acetylated
KAP104	MAS	cleaved	Acetylated
KAR3	ME	uncleaved	Acetylated
KCS1	MD	uncleaved	Acetylated
KIN2	MP	cleaved	non-acetylated
KIP1	MAR	cleaved	non-acetylated
KSP1	MT	cleaved	non-acetylated
LEU2	MS	cleaved	Acetylated
LIA1	MS	cleaved	Acetylated
LIA1	MS	cleaved	Acetylated
LSM1	MS	cleaved	Acetylated
LSM2	MLF	uncleaved	non-acetylated
LTP1	MT	cleaved	non-acetylated
LYS2	MT	cleaved	non-acetylated
LYS5	MV	cleaved	non-acetylated
LYS9	MG	cleaved	non-acetylated
MAP1	MS	cleaved	Acetylated
MAP2	MT	cleaved	non-acetylated
MCM2	MS	cleaved	Acetylated
MDY2	MS	cleaved	Acetylated
MES1	MS	cleaved	Acetylated
MET1	MV	cleaved	non-acetylated
MET14	MAT	cleaved	non-acetylated
MET6	MV	cleaved	non-acetylated
MET8	MV	cleaved	non-acetylated
MIH1	MN	uncleaved	Acetylated *
MON2	MAM	cleaved	non-acetylated
MOT2	MMN	uncleaved	Acetylated
MUQ1	MT	cleaved	non-acetylated
MYO2	MS	cleaved	Acetylated
MYO4	MS	cleaved	Acetylated
NAR1	MS	cleaved	Acetylated
NCS2	ME	uncleaved	Acetylated
NMD3	ME	uncleaved	Acetylated
NPL4	MLI	uncleaved	non-acetylated
NRK1	MT	cleaved	non-acetylated
OPI3	MK	uncleaved	non-acetylated
PAB1	MAD	cleaved	non-acetylated
PAC10	MD	uncleaved	Acetylated
PAN1	MY	uncleaved	non-acetylated
PCK1	MS	cleaved	Acetylated
PCT1	MAN	cleaved	non-acetylated

PFD1	MS	cleaved	Acetylated
PFK1	MQ	uncleaved	non-acetylated
PFK27	MG	cleaved	non-acetylated
PFY1	MS	cleaved	Acetylated
PGI1	MS	cleaved	Acetylated
PIG2	MAT	cleaved	non-acetylated
PKC1	MS	cleaved	Acetylated
PPG1	ME	uncleaved	Acetylated
PPQ1	MR	uncleaved	non-acetylated
PPT1	MS	cleaved	Acetylated
PPZ1	MG	cleaved	non-acetylated
PRE2	MQ	uncleaved	non-acetylated
PRE6	MS	cleaved	Acetylated
PRR1	MD	uncleaved	Acetylated
PRS1	MR	uncleaved	non-acetylated
PTC1	MS	cleaved	Acetylated
PTP3	MK	uncleaved	non-acetylated
PUF2	MD	uncleaved	Acetylated
PYC1	MS	cleaved	Acetylated
PYC2	MS	cleaved	Acetylated
RAD6	MS	cleaved	Acetylated
RAS1	MQ	uncleaved	non-acetylated
RBL2	MAP	cleaved	non-acetylated
RFA1	MS	cleaved	Acetylated
RGA1	MAS	cleaved	Acetylated
RIM13	MN	uncleaved	Acetylated *
RNR4	ME	uncleaved	Acetylated
RPL17A	MAR	cleaved	non-acetylated
RPL19B	MAN	cleaved	non-acetylated
RPL23A	MS	cleaved	Acetylated
RPL39	MAA	cleaved	non-acetylated
RPL4A	MS	cleaved	Acetylated
RPN1	MV	cleaved	non-acetylated
RPN2	MS	cleaved	Acetylated
RPS3	MV	cleaved	non-acetylated
RPS6A	MK	uncleaved	non-acetylated
RPS7B	MS	cleaved	Acetylated
RPS8A	MG	cleaved	non-acetylated
RTG3	MMN	uncleaved	Acetylated
RVS161	MS	cleaved	Acetylated
RVS167	MS	cleaved	Acetylated
SAC6	MN	uncleaved	Acetylated *
SAC7	MP	cleaved	non-acetylated
SAR1	MAG	cleaved	non-acetylated
SCP160	MS	cleaved	Acetylated
SEC13	MV	cleaved	non-acetylated
SEC31	MV	cleaved	non-acetylated
SER1	MS	cleaved	Acetylated
SER2	MS	cleaved	Acetylated
SES1	MLD	uncleaved	non-acetylated
SHE2	MS	cleaved	Acetylated
SHE3	MS	cleaved	Acetylated
SHM2	MP	cleaved	non-acetylated

SIC1	MT	cleaved	non-acetylated
SIT4	MV	cleaved	non-acetylated
SIW14	MG	cleaved	non-acetylated
SIZ1	MI	uncleaved	non-acetylated
SKO1	MS	cleaved	Acetylated
SLA1	MT	cleaved	non-acetylated
SMY1	MHW	uncleaved	non-acetylated
SRP1	MD	uncleaved	Acetylated
SRP101	MFD	uncleaved	non-acetylated
SRP14	MAN	cleaved	non-acetylated
SRP54	MV	cleaved	non-acetylated
SSA1	MS	cleaved	Acetylated
SSB1	MAE	cleaved	Acetylated
STI1	MS	cleaved	Acetylated
STU1	MS	cleaved	Acetylated
SUI2	MS	cleaved	Acetylated
SUP35	MS	cleaved	Acetylated
SUP45	MD	uncleaved	Acetylated
SYN8	MD	uncleaved	Acetylated
TEF1	MG	cleaved	non-acetylated
TEF2	MG	cleaved	non-acetylated
TIF1	MS	cleaved	Acetylated
TMA46	MP	cleaved	non-acetylated
TMT1	MS	cleaved	Acetylated
TPD3	MS	cleaved	Acetylated
TPM1	MD	uncleaved	Acetylated
TRP1	MS	cleaved	Acetylated
TSA1	MV	cleaved	non-acetylated
TUB1	MR	uncleaved	non-acetylated
TUB2	MR	uncleaved	non-acetylated
UBA1	MS	cleaved	Acetylated
UBP6	MS	cleaved	Acetylated
UFD1	MFS	uncleaved	non-acetylated
URA3	MS	cleaved	Acetylated
URA7	MK	uncleaved	non-acetylated
URA8	MK	uncleaved	non-acetylated
UTR1	MK	uncleaved	non-acetylated
VID30	MS	cleaved	Acetylated
VPS1	MD	uncleaved	Acetylated
YMR099c	MP	cleaved	non-acetylated
YVH1	MAG	cleaved	non-acetylated
ZUO1	MFS	uncleaved	non-acetylated
ZWF1	MS	cleaved	Acetylated

* MN acetylation is predicted in ony 55% of cases
