# Supplementary material for: N-Terminal Acetylation Inhibits Protein Targeting to the Endoplasmic Reticulum
Source: PLoS Biol. 2011 May 31;9(5):e1001073. doi: 10.1371/journal.pbio.1001073 (PMC3104963; doi:10.1371/journal.pbio.1001073)
Supplement: Table S3 — Relative amino acid frequency at position 2 by compartment in yeast. (PDF) [file pbio.1001073.s008.pdf]

Table S3: Relative Amino Acid Frequency at Position 2 by Compartment in Yeast

| P2 Residue | Signal Sequence | Cytosol | f(ss)/f(cyt) |
|---|---|---|---|
| A | 3.25 | 10.32 | 0.317 |
| C | 0.00 | 0.00 | ∞ |
| D | 0.72 | 6.75 | 0.108 |
| E | 0.72 | 4.37 | 0.167 |
| F | 6.50 | 1.19 | 4.887 |
| G | 0.72 | 5.56 | 0.131 |
| H | 2.17 | 0.79 | 2.749 |
| I | 6.86 | 0.79 | 8.705 |
| K | 16.61 | 3.17 | 5.269 |
| L | 13.72 | 3.17 | 4.353 |
| M | 1.44 | 1.19 | 1.222 |
| N | 3.61 | 2.38 | 1.527 |
| P | 1.44 | 5.56 | 0.262 |
| Q | 10.11 | 1.98 | 5.132 |
| R | 13.00 | 1.98 | 6.598 |
| S | 5.78 | 35.32 | 0.165 |
| T | 4.33 | 7.54 | 0.579 |
| V | 7.58 | 7.54 | 1.013 |
| W | 1.08 | 0.00 | ∞ |
| Y | 0.36 | 0.40 | 0.916 |
| Total | 100.00 | 100.00 | |
| n | 277 | 251 | |
