# Supplementary material for: N-Terminal Acetylation Inhibits Protein Targeting to the Endoplasmic Reticulum
Source: PLoS Biol. 2011 May 31;9(5):e1001073. doi: 10.1371/journal.pbio.1001073 (PMC3104963; doi:10.1371/journal.pbio.1001073)
Supplement: Table S4 — Predicted relative frequency of N-terminal methionine cleavage. (PDF) [file pbio.1001073.s009.pdf]

Table S4: Predictied Relative Frequency of N-terminal methionine cleavage

	Signal Sequence	Cytosol
cleaved	23.1	71.8
uncleaved	76.9	28.2
