# Supplementary material for: N-Terminal Acetylation Inhibits Protein Targeting to the Endoplasmic Reticulum
Source: PLoS Biol. 2011 May 31;9(5):e1001073. doi: 10.1371/journal.pbio.1001073 (PMC3104963; doi:10.1371/journal.pbio.1001073)
Supplement: Table S5 — Relative P2 frequency of signal sequences from different organisms. (PDF) [file pbio.1001073.s010.pdf]

Table S5 Relative P2 Frequency of Signal Sequences from different Organisms

P2 residue	Human	C. elegans	S. cerevisiae	D.melanogaster	A.thaliana
A	18.99	1.59	2.94	7.81	25.00
C	0.34	1.85	0.00	0.89	0.40
D	1.85	1.06	1.10	1.34	3.57
E	5.04	1.32	1.10	2.23	7.74
F	2.02	4.23	6.62	6.47	0.79
G	9.41	2.12	0.74	4.02	8.33
H	1.01	2.65	2.21	2.01	0.40
I	3.03	7.41	6.62	2.46	1.39
K	9.92	12.96	16.18	18.08	18.45
L	8.07	7.94	11.40	8.48	3.17
M	1.51	2.38	1.84	3.57	2.78
N	1.68	8.47	4.78	6.70	2.98
P	2.86	2.65	1.10	3.57	0.79
Q	3.70	4.23	11.76	4.91	0.99
R	13.28	23.54	13.24	10.04	6.15
S	5.21	5.82	5.51	7.14	8.53
T	2.69	5.56	4.04	3.57	4.37
V	6.05	1.85	7.72	2.46	3.57
W	2.18	1.32	0.74	2.01	0.20
Y	1.18	1.06	0.37	2.23	0.40
n	595	378	277	448	500
