# Supplementary material for: N-Terminal Acetylation Inhibits Protein Targeting to the Endoplasmic Reticulum
Source: PLoS Biol. 2011 May 31;9(5):e1001073. doi: 10.1371/journal.pbio.1001073 (PMC3104963; doi:10.1371/journal.pbio.1001073)
Supplement: Table S6 — Predicted frequency of N-terminal processing of signal sequences from different organisms. (PDF) [file pbio.1001073.s011.pdf]

Table S6 Predicted Frequency of N-terminal processing of Signal Sequences from different organisms

	Predicted N-terminal Processing				
Organism	Unprocessed	Met-cleaved	Uncleaved & acetylated	Met-cleaved & acetylated	n
S. ceverevisiae	66	16	11	7	277
C. elegans	68	15	11	6	378
D. melanogaster	60	17	10	13	448
Human	46	23	23	8	595
A. thaliana	34	24	14	28	500
