# Supplementary material for: N-Terminal Acetylation Inhibits Protein Targeting to the Endoplasmic Reticulum
Source: PLoS Biol. 2011 May 31;9(5):e1001073. doi: 10.1371/journal.pbio.1001073 (PMC3104963; doi:10.1371/journal.pbio.1001073)
Supplement: Table S7 — Yeast strains used in this study. (PDF) [file pbio.1001073.s012.pdf]

Table S7 ***Yeast strains used in this study***

**Name**	*Genotype*	**Reference**
MWY63	*Mat***a** *prc1::Kan*MX *leu2 his3 ura3 ade2 pep4-3 sec61-3*	[1]
Δprc1	*Matα prc1::KanMX4 his3Δ1 leu2Δ0 lys2Δ0 ura3Δ0*	[2]
Δpep4	*Mat**a** pep4::KanMX4 his3Δ1 leu2Δ0 met15Δ0 ura3Δ0*	[2]
GFY3	*Mat**a** prc1::KanMX4 pep4:: KanMX4 his3Δ1 leu2Δ0 ura3Δ0*	This study
GFY7	*Mat**a** prc1::KanMX4 pep4:: KanMX4 map1::HIS3MX6 his3Δ1 leu2Δ0 ura3Δ0*	This study
GFY11	*Matα prc1::KanMX4 ard1::hph his3Δ1 leu2Δ0 lys2Δ0 ura3Δ0*	This study
GFY12	*Matα prc1::KanMX4 nat3:: hph his3Δ1 leu2Δ0 lys2Δ0 ura3Δ0*	This study

References

[1] Willer M, Forte GM, Stirling CJ (2008) Sec61p is required for ERAD-L: genetic dissection of the translocation and ERAD-L functions of Sec61p using novel derivatives of CPY. J Biol Chem. 283: 33883-33888.

[2] Winzeler EA, Shoemaker DD, Astromoff A, Liang H, Anderson K, et al. (1999) Functional characterization of the *S. cerevisiae* genome by gene deletion and parallel analysis. Science 285: 901-906.
