# Supplementary material for: N-Terminal Acetylation Inhibits Protein Targeting to the Endoplasmic Reticulum
Source: PLoS Biol. 2011 May 31;9(5):e1001073. doi: 10.1371/journal.pbio.1001073 (PMC3104963; doi:10.1371/journal.pbio.1001073)
Supplement: Table S8 — Oligonucleotides used in this study. (PDF) [file pbio.1001073.s013.pdf]

Table S8 ***Oligonucleotides used in this study***

Name	Sequence	Description
Map1 KO F	TCCTAGCAAGAAAAAAATAAGCAAAAAAATTGTATAATCGGATCCCCGGGTTAATTAA	*HIS3MX6* disruption *MAP1*
Map1 KO R	GTACAAGTTCAAGTTTTTTATTGGTTTCTATATGTATAAGATAAGAATTCGAGCTCGTTTAAAC	*HIS3MX6* disrupt *MAP1*
Ard1/A KO F2	AAATACATACGATCAAGCTCCAAAATAAACTTCGTCAACCCGGATCCCCGGGTTAATTAA	Hph disruption *ARD1*
Ard1/A KO R	CCTGGATGAAAATATACTACGTTTATATAGGTTGATTTAAAATACGACTCACTATAGGGAG	Hph disruption *ARD1*
Nat3/B KO F2	ATTGAGAATATTCAAGGAAAGAGACAGGAGGATTCGAGAACGGATCCCCGGGTTAATTAA	Hph disruption *NAT3*
Nat3/B KO R	ATTATTATGTTCTGAGTATGAGGACGAGGTAATACATACCAATACGACTCACTATAGGGAG	Hph disruption *NAT3*
CPY-A-F	CAACTTAAAGTATACATACGCTATGGCTAAAGCATTCACCAGTTTACTATG	SDM of pMW346
CPY-A-R	CATAGTAAACTGGTGAATGCTTTAGCCATAGCGTATGTATACTTTAAGTTG	"
CPY-C-F	CAACTTAAAGTATACATACGCTATGTGTAAAGCATTCACCAGTTTACTATG	"
CPY-C-R	CATAGTAAACTGGTGAATGCTTTACACATAGCGTATGTATACTTTAAGTTG	"
CPY-E-F	CAACTTAAAGTATACATACGCTATGGAAAAAGCATTCACCAGTTTACTATG	"
CPY-E-R	CATAGTAAACTGGTGAATGCTTTTTCCATAGCGTATGTATACTTTAAGTTG	"
CPY-G-F	CAACTTAAAGTATACATACGCTATGGGTAAAGCATTCACCAGTTTACTATG	"
CPY-G-R	CATAGTAAACTGGTGAATGCTTTACCCATAGCGTATGTATACTTTAAGTTG	"
CPY-R-F	CAACTTAAAGTATACATACGCTATGAGAAAAGCATTCACCAGTTTACTATG	"
CPY-R-R	CATAGTAAACTGGTGAATGCTTTTCTCATAGCGTATGTATACTTTAAGTTG	"
CPY-S-F	CAACTTAAAGTATACATACGCTATGTCCAAAGCATTCACCAGTTTACTATG	"
CPY-S-R	CATAGTAAACTGGTGAATGCTTTGGACATAGCGTATGTATACTTTAAGTTG	"
CPY-V-F	CAACTTAAAGTATACATACGCTATGGTCAAAGCATTCACCAGTTTACTATG	"
CPY-V-R	CATAGTAAACTGGTGAATGCTTTGACCATAGCGTATGTATACTTTAAGTTG	"
OPY-A-F	GGTGCTGAAAAAATGGCTAGGCAGGTTTGGTTC	SDM of pOPY
OPY-A-R	GAACCAAACCTGCCTAGCCATTTTTTCAGCACC	"
OPY-C-F	GGTGCTGAAAAAATGTGTAGGCAGGTTTGGTTC	"
OPY-C-R	GAACCAAACCTGCCTACACATTTTTTCAGCACC	"
OPY-E-F	GGTGCTGAAAAAATGGAAAGGCAGGTTTGGTTC	"
OPY-E-R	GAACCAAACCTGCCTTTCCATTTTTTCAGCACC	"
OPY-G-F	GGTGCTGAAAAAATGGGTAGGCAGGTTTGGTTC	"
OPY-G-R	GAACCAAACCTGCCTACCCATTTTTTCAGCACC	"
OPY-S-F	TTGGTGCTGAAAAAATGTCTAGGCAGGTTTGGTTCTCTTGG	"
OPY-S-R	CCAAGAGAACCAAACCTGCCTAGACATTTTTTCAGCACCAA	"
PDI-F	ACTTAAAGTTATAACATACGCTATGAAGTTTTCTGCTGGTGCCGTC	Myc-tagging PDI1
PDI-S-F	ACTTAAAGTTATAACATACGCTATGTCTAAGTTTTCTGCTGGTGCCGTC	SDM of pPDI1-myc
PDI-E-F	ACTTAAAGTTATAACATACGCTATGGAGAAGTTTTCTGCTGGTGCCGTC	"
PDI-myc-R	ACCACCGTGGATCCATTCCTACAAATCTTCTTCAGAAATCAATTTTTGTTCCAATTCATCGTGAA TGGCATC	Myc-tagging PDI1
PPαF-F	CAACTTAAAGTATACATACGCTATGAGATTTCCTTCAAT	Myc-tagging ppαF
PPαF-S-F	CAACTTAAAGTATACATACGCTATGTCTAGATTTCCTTCAAT	SDM of ppAF-2myc
PPαF-2myc-R	ACCACCGTGGATCCATTCTTACAAATCTTCTTCAGAAATCAATTTTTGTTCCAGGTCCTCCTCG CTGATCAGCTTCTGCTCGTACATTGGTTGGCCGGG	Myc-tagging ppαF
ppαF wt sp6 F	ATTTAGGTGACACTATAGACGATTAAAAGAATGAGATTTC	MR mRNA template
ppαF S sp6 F	ATTTAGGTGACACTATAGACGATTAAAAGAATGTCCAGATTTCCTTCAAT	MSR mRNA template
ppαF msrr F	ATTTAGGTGACACTATAGACGATTAAAAGAATGTCCAGAAGATTTCCTTC	MSRR mRNA template
pEH3 R	GCCTGCAGGTCGACTCTAGAGTCG	pEH3 mRNA template
ppalpha k5 R	GCATGCCTGCAGGTCGACTTTGTTAC	pGF22 mRNA template
Ost1 ss EcoRI-f	GCAAGCTGAATTCCTTCTTTGACAAGTACCCGATTGC	PCR OST1 signal sequence

Ost1 ss HincII-r	GGACAGGATGTTGACCGGCTCGTATTGGGCAGCAG	PCR OST1 signal sequence
DHC sp6 wt F	ATTTAGGTGACACTATAGAAGAGGATCCGGATCCATGAGAG	MR mRNA template
DHC sp6 S F	ATTTAGGTGACACTATAGAAGAGGATCCGGATCCATGTCAAGAGTCGGTATTATCTTCG	MSR mRNA template
