# Supplementary material for: N-Terminal Acetylation Inhibits Protein Targeting to the Endoplasmic Reticulum
Source: PLoS Biol. 2011 May 31;9(5):e1001073. doi: 10.1371/journal.pbio.1001073 (PMC3104963; doi:10.1371/journal.pbio.1001073)
Supplement: Table S9 — Plasmids used in this study. (PDF) [file pbio.1001073.s014.pdf]

Table S9 ***Plasmids used in this study***

Name	Description	Source
pMW346	ppCPY *LEU2* pRS315	[1]
pCPY-A	SDM of pMW346 Alanine inserted at position 2 of signal sequence	This study
pCPY-C	SDM of pMW346 Cysteine inserted at position 2 of signal sequence	This study
pCPY-E	SDM of pMW346 Glutamate inserted at position 2 of signal sequence	This study
pCPY-G	SDM of pMW346 Glycine inserted at position 2 of signal sequence	This study
pCPY-R	SDM of pMW346 Arginine inserted at position 2 of signal sequence	This study
pCPY-S	SDM of pMW346 Serine inserted at position 2 of signal sequence	This study
pCPY-V	SDM of pMW346 Valine inserted at position 2 of signal sequence	This study
pOPY	pMW346 modified so that Ost1 signal sequence replaced that of CPY	This study
pOPY-A	SDM of pOPY Alanine inserted at position 2 of signal sequence	This study
pOPY-C	SDM of pOPY Cysteine inserted at position 2 of signal sequence	This study
pOPY-E	SDM of pOPY Glutamate inserted at position 2 of signal sequence	This study
pOPY-G	SDM of pOPY Glycine inserted at position 2 of signal sequence	This study
pOPY-S	SDM of pOPY Serine inserted at position 2 of signal sequence	This study
pPDI-myc	pMW346 with CPY ORF replaced with C-terminal myc-tagged *PDI1*	This study
pPDI-myc-S	pMW346 with CPY ORF replaced with C-terminal myc-tagged *PDI1*-MS mutant	This study
pPDI-myc-E	pMW346 with CPY ORF replaced with C-terminal myc-tagged *PDI1*-ME mutant	This study
pPPαF-2myc	pMW346 with CPY ORF replaced with C-terminal 2xmyc-tagged ppαF	This study
pPPαF-2myc-S	pMW346 with CPY ORF replaced with C-terminal 2xmyc-tagged ppαF ME mutant	This study
pA11-K5K14	K5 ppαF in pAlter, all lysines codons in *wild-type* ppαF altered to arginine and lysines introduced at positions 5 and 14 of signal sequence	[2]
pEH3	pGEM3-ppαF	[3]
pGF22	pGEM3-ppαF with all lysines codons in *wild-type* ppαF altered to arginine	This study
pGF23	pGF22 cut SalI klenow filled and relegated to remove one HincII site	This study
pGF24	O-αFactor pGF22 signal sequence of ppαF replaced with that of OST1	This study
pGF25	As pGF24 except for insertion of serine at position 2 of OST1 signal sequence	This study
pGF28	As for pGF22 but with the signal sequence replaced by that of D_{HC} from pJD96	This study
pJD96	p-D_{hc}αF (codon-bias optimised)	[4]

References

[1] Willer M, Forte GM, Stirling CJ (2008) Sec61p is required for ERAD-L: genetic dissection of the translocation and ERAD-L functions of Sec61p using novel derivatives of CPY. J Biol Chem. 283: 33883-33888.

[2] Plath K, Mothes W, Wilkinson BM, Stirling CJ, Rapoport TA (1998) Signal sequence recognition in posttranslational protein transport across the yeast ER membrane. Cell 94: 795-807.

[3] Steel GJ, Brownsword J, Striling CJ (2002) Tail-Anchored Protein Insertion into Yeast ER requires Novel a Posttranslational Mechanism Which is Independent of the SEC Machinery. Biochemistry 41: 11914-11920.

[4] Mason N, Ciufo LF, Brown JD (2000) Elongation arrest is a physiologically important function of the signal recognition particle. EMBO J. 19: 4164-4174.
