# Supplementary material for: N-Terminal Acetylation Inhibits Protein Targeting to the Endoplasmic Reticulum
Source: PLoS Biol. 2011 May 31;9(5):e1001073. doi: 10.1371/journal.pbio.1001073 (PMC3104963; doi:10.1371/journal.pbio.1001073)
Supplement: Text S1 — Supporting methods. (PDF) [file pbio.1001073.s015.pdf]

Supporting Methods

Sample preparation

Wild-type *(GFY3)* yeast harbouring plasmid pPdi1myc-S were grown at 30°C in 200 mL minimal media to an OD_{600} of ≈1.8. Cells were washed with dH_2O and then lysed with glass beads in 2 mL of ice cold lysis buffer (50 mM Hepes-KOH pH 7.6, 1 mM EDTA, 0.1 % [v/v] Triton X-100, 0.2 M KCl, 5 mM 2-mercaptoethanol, 10% [v/v] glycerol, 10 $\mu g.mL^{-1}$ leupeptin, 10 $\mu g.mL^{-1}$ pepstatin, 0.5 mM PMSF, 1X complete EDTA-free protease inhibitor pill [Roche]). Cellular debris and glass beads were removed by centrifugation (2min, 1200 *g,* 4°C) and then the extracts cleared by further centrifugation (20 min, 16000 *g,* 4°C). Extracts were then preadsorbed for 30 min with 150 μL insoluble Protein A (Sigma), and then rotated with 30 μg anti-myc antibodies (Clone 4A6, Millipore) prebound to 60 μL Protein G-sepharose (Zymed) for 3h in the cold. Beads were then washed twice with lysis buffer, twice with 20 mM Tris-HCl pH7.6, 500 mM NaCl, 0.4% (v/v) NP-40 and once with 10 mM Tris-HCl pH 7.6 prior to elution with SDS-PAGE sample buffer. Eluted material was then separated on a 7.5% Tris-glycine SDS-PAGE gel, stained with Instant Blue (Expedeon) and the MS-pPdi1p-myc precursor band excised. The sample was reduced, alkylated with iodoacetamide, and then digested overnight at 37°C with elastase.

Mass-spectrometry

Digested samples were analysed by LC-MS/MS using a NanoAcquity LC (Waters, Manchester, UK) coupled to a LTQ Velos (Thermo Fisher Scientific, Waltham, MA). Peptides were concentrated on a pre-column (20 mm x 180 µm i.d, Waters). The peptides were then separated using a gradient from 99% A (0.1% formic acid in water) and 1% B (0.1% formic acid in acetonitrile) to 30% B, in 40 min at 300 $nL.min^{-1}$, using a 75 mm x 250 µm i.d. 1.7 μM BEH C18, analytical column (Waters). Peptides were selected for fragmentation automatically by data-dependant analysis.

Data analysis

Peak lists were generated from the data using the MSN_Extract utility via Mascot Daemon software (Matrix Science, UK). The peak-list file was edited to include four spectra from a previous trypsin digestion and searched against a yeast-specific database with trypsin as the enzyme and fixed carbamidomethyl modification of cysteines using Mascot (v 2.2, Matrix Science, UK). The additional spectra allowed Mascot to identify PDI and the data was then resubmitted using the error-tolerant option required for matching elastase-generated data. Acquired data were further validated using Scaffold (v 3.0, Proteome Software, Portland, OR). Matches were considered significant if they had greater than 95.0% probability as specified by the Peptide Prophet algorithm[1]. Matches were further validated by manual inspection to compare between the highest match and the nearest alternative.

Reference

1. Keller A, Nesvizhskii AI, Kolker E, Aebersold R (2002) Empirical statistical model to estimate the accuracy of peptide identifications made by MS/MS and database search. Anal Chem 74: 5383-5392.
